# Mesenchyme-derived inflammation during the saccular stage recruits macrophages and alters lung development

**DOI:** 10.1172/jci.insight.193625

**Published:** 2026-06-02

**Authors:** Benjamin C. Crawford, Jessica Chauviere Lee, Bertha C. Elias, Shivangi Dave, Riet van der Meer, Wei Han, Alexandria L. Sharkey, David S. Nichols, Charles Shissias, Lauren Pate, Hayden Tan, Dawn C. Newcomb, Wei Shi, Lawrence S. Prince, Erin J. Plosa, Bradley W. Richmond, Timothy S. Blackwell, Susan H. Guttentag, John T. Benjamin

**Affiliations:** 1 Department of Pediatrics, Division of Neonatology, and; 2 Department of Medicine, Division of Allergy, Pulmonary, and Critical Care Medicine, Vanderbilt University Medical Center, Nashville, Tennessee, USA.; 3 Department of Internal Medicine, Division of Critical Care and Pulmonary Medicine, University of Michigan Medical School, Ann Arbor, Michigan, USA.; 4 Department of Internal Medicine, Division of Pulmonary, Critical Care and Sleep Medicine, University of Cincinnati College of Medicine, Cincinnati, Ohio, USA.; 5 Department of Pediatrics, Division of Neonatal and Developmental Medicine, Stanford University School of Medicine, Palo Alto, California, USA.; 6 Department of Pediatrics, Division of Neonatal-Perinatal Medicine, University of North Carolina at Chapel Hill, Chapel Hill, North Carolina, USA.; 7 Department of Veterans Affairs Medical Center, Nashville, Tennessee, USA.

**Keywords:** Development, Inflammation, Pulmonology, Endothelial cells, Macrophages, NF-kappaB

## Abstract

Fibroblasts in the lung mesenchyme produce growth factors and extracellular matrix components that guide formation of distal airspaces during the saccular stage of lung development. Inflammation in preterm infants disrupts this process, leading to bronchopulmonary dysplasia (BPD). To examine how mesenchymal inflammation contributes to BPD pathogenesis, we developed a transgenic mouse model (IKKβ^Tbx4^) in which expression of activated human IκB kinase β (IKKβ), an upstream activator of NF-κB, was induced in *Tbx4* lung enhancer–positive mesenchymal cells during the saccular stage of lung development (P0–P5). Saccular-stage IKKβ^Tbx4^ mice exhibited a BPD-like phenotype with interstitial thickening and reduced distal airspaces at P5, progressing to emphysematous enlargement of the distal lung at 2 months of age. Mesenchymal NF-κB activity upregulated the chemokines CCL2 and CCL7, recruiting CCR2^pos^ monocyte-derived macrophages to the lung. Recruited macrophages disrupted the elastin scaffold and impaired microvascular organization with reductions in CAP2 endothelial cells and pericytes. Blocking CCR2-dependent monocyte recruitment with a small-molecule CCR2 antagonist rescued the abnormal lung phenotype. These findings identify mesenchyme-macrophage crosstalk as a mechanism by which inflammation disrupts saccular-stage lung development, suggesting a role for this signaling axis in BPD pathogenesis.

## Abstract 2

Inflammatory signaling in saccular stage mouse lung mesenchyme recruits macrophages that disrupt lung development, leading to sustained structural and functional deficits resembling a BPD lung.

## Graphical abstract

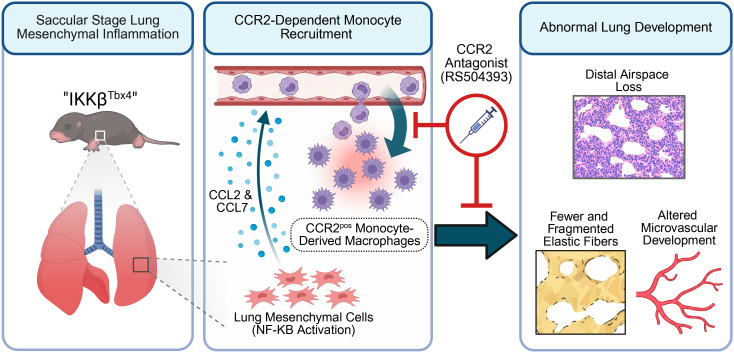

## Introduction

The saccular stage of lung development marks a critical period during which lung mesenchymal cells guide the formation of primitive air sacs in the distal lung. During sacculation, fibroblasts produce growth factors and extracellular matrix components that direct epithelial and endothelial organization and provide the structural support for alveolar development (1–3). Disrupting these critical events can permanently alter the form and function of the developing lung and lead to bronchopulmonary dysplasia (BPD) (4–8), a chronic respiratory condition that primarily affects preterm infants born before 30 weeks of gestation (9). BPD is characterized by alveolar simplification and dysmorphic lung structure (10–15). Clinically, BPD is defined by an extended need for respiratory support, and affected individuals are at increased risk of pulmonary impairment throughout life (16–18). Despite improvements in neonatal clinical care over the past several decades, effective therapeutic options for affected infants are still lacking, and the incidence of BPD has continued to increase (19, 20).

Both clinical and laboratory data demonstrate that antenatal and early postnatal inflammation can alter saccular-stage lung development and contribute to BPD. In utero exposure to chorioamnionitis is an independent risk factor for developing BPD (21–23). Further, early life exposures that cause pulmonary inflammation in preterm infants, such as mechanical ventilation and oxygen supplementation, can injure the lung and disrupt its development (24–29). Animal studies corroborate these findings, demonstrating that inflammation in the immature lung leads to a BPD-like phenotype (30–34). However, the mechanisms linking inflammatory signaling to the molecular events mediating abnormal lung morphogenesis remain unclear.

In addition to their well-described role in lung organogenesis, cells of the distal lung mesenchyme also participate in immune signaling. Multiple mesenchymal subpopulations express TLRs and other pathogen-recognition receptors that activate inflammation via the NF-κB pathway (35–38). Upon activation by inflammatory stimuli, the lung mesenchyme produces chemokines and cytokines that amplify inflammatory signaling and recruit immune cells to the lung (39–44). Recent transcriptomic profiling of BPD models has identified proinflammatory populations of fibroblasts in the lung mesenchyme (34, 45, 46). Predicted to function as central hubs of paracrine signaling, inflammatory fibroblasts exhibit robust chemokine expression that correlates with immune cell recruitment. Yet, whether inflammation originating from the lung mesenchyme is sufficient to disrupt saccular-stage development has not been tested.

To examine the cell-specific effects of mesenchymal inflammation on the developing postnatal lung, we generated a transgenic mouse model (IKKβ^Tbx4^) in which we induced expression of IκB kinase β (IKKβ) in Tbx4 lung enhancer–expressing mesenchymal cells during the saccular stage of lung development (P0–P5). We demonstrated that mesenchymal-derived inflammation during this critical developmental window was sufficient to recruit CCR2^pos^ monocyte-derived macrophages and alter distal lung structure. Our findings establish that mesenchymal-macrophage crosstalk disrupts saccular-stage development, leading to characteristic features observed in the BPD lung.

## Results

### Mesenchymal inflammation alters saccular-stage lung development.

**Figure 1:**
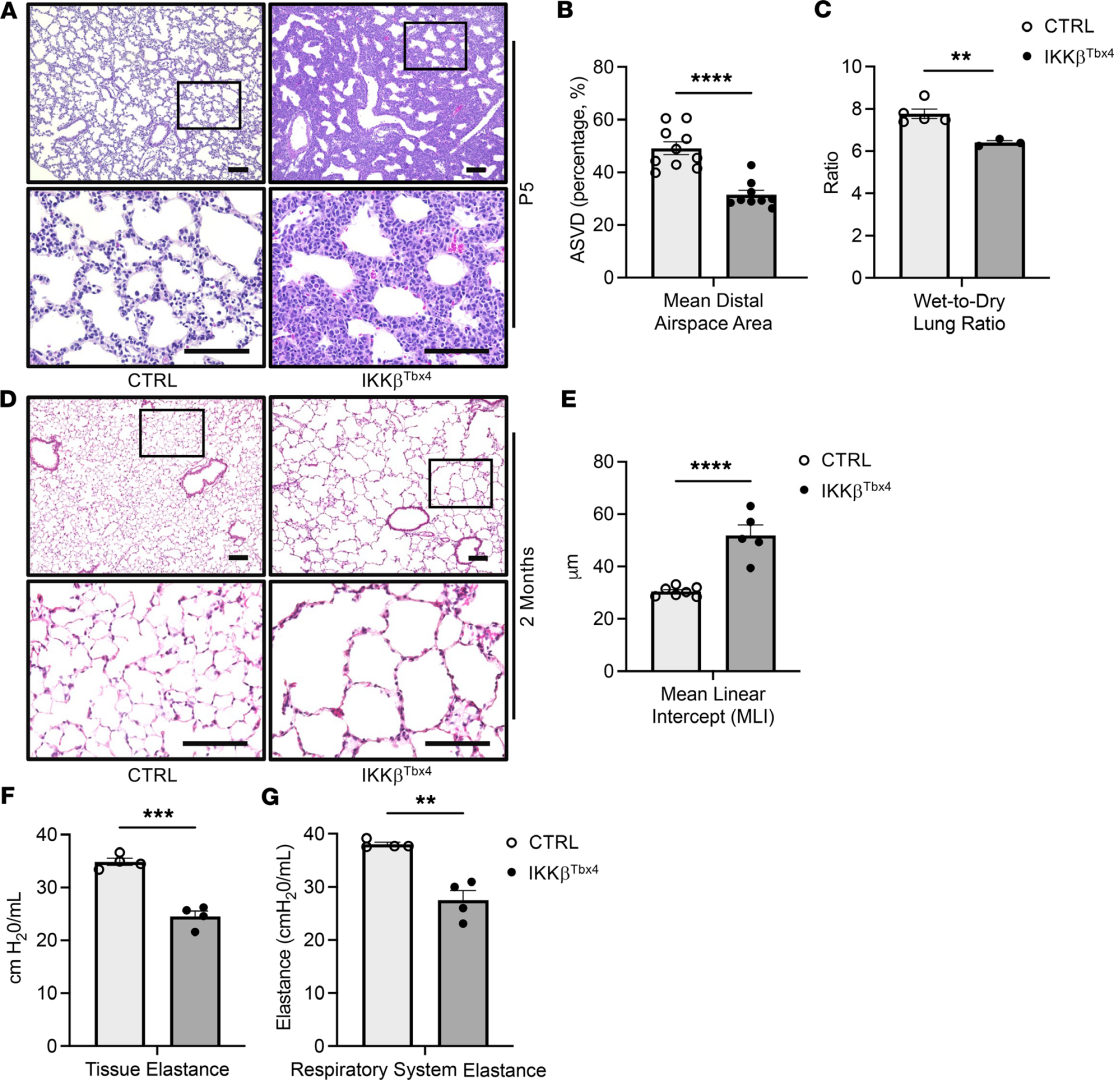
NF-κB activation in the lung mesenchyme alters saccular-stage lung development. Doxycycline was added to drinking water of lactating dams from P0 to P5, and neonatal lungs were harvested on P5 (**A** and **B**) or at 2 months of age (**D**–**G**). (**A** and **D**) Representative H&E-stained lung sections of littermate control (CTRL) and IKKβ^Tbx4^ mice at P5 (**A**) and 2 months of age (**D**). (**B** and **E**) Morphometric quantification of airspace volume density (ASVD) at P5 (**B**) and mean linear intercept (MLI) at 2 months of age (**E**). (**C**) Wet-to-dry lung weight ratios in CTRL and IKKβ^Tbx4^ lungs at P5. (**F** and **G**) Lung tissue (**F**) and respiratory system (**G**) elastance were measured at 2 months of age using flexiVent in CTRL and IKKβ^Tbx4^ mice. Data are expressed as mean ± SEM. *n* = 10 to 12 nonoverlapping lung fields per lung (biological replicate) (**B** and **E**); *n* = 9 to 10 mice per group (**B**); *n* = 3 to 5 mice per group (**C**); *n* = 5 to 7 mice per group (**E**); and *n* = 4 mice per group (**F** and **G**); scale bar: 100 μm (**A** and **D**); **P < 0.01, ***P < 0.001, and ****P < 0.0001 by unpaired 2-tailed *t* test (**B**, **C**, and **E**–**G**).

We developed a transgenic mouse model (IKKβ^Tbx4^) to investigate the effects of mesenchymal inflammation on the developing saccular-stage lung. In this model, an activated form of human IKKβ, the upstream activator of NF-κB, is expressed in cells with *Tbx4* lung enhancer activity in a doxycycline-inducible (Dox-inducible) manner. The breeding strategy used to generate IKKβ^Tbx4^ and littermate control mice is shown in Supplemental Figure 1A (supplemental material available online with this article; https://doi.org/10.1172/jci.insight.193625DS1). Lactating dams were administered Dox from P0 to P5 (Supplemental Figure 1B) to selectively induce expression of the IKKβ transgene and activate NF-κB in the lung mesenchyme of transgenic pups (Supplemental Figure 2, A–C). In littermate control mice, the distal lung parenchyma demonstrated arborized and well-distributed distal airspaces separated by a thin interstitium. In comparison, lungs from Dox-exposed IKKβ^Tbx4^ mice were markedly hypercellular with interstitial thickening and a significant reduction in distal airspace area (Figure 1, A and B). These morphological changes were not attributable to pulmonary edema, as the wet-to-dry lung weight ratio was not increased in transgenic mice compared with controls (Figure 1C).

Despite the severe structural abnormalities at P5, IKKβ^Tbx4^ mice survived into adulthood but exhibited long-term consequences from neonatal inflammation. Adult IKKβ^Tbx4^ lungs appeared emphysematous, with markedly enlarged distal airspaces compared with littermate controls (Figure 1, D and E). Consistent with this structural phenotype, IKKβ^Tbx4^ mice demonstrated impaired lung mechanics, with reduced tissue and total lung elastance (Figure 1, F and G). Transgene induction was required for these abnormalities, as Dox-naive IKKβ^Tbx4^ mice and Dox-exposed littermate controls exhibited normal lung morphology at both P5 and 2 months of age (Supplemental Figure 3, A–D).

To identify the mesenchymal cell populations targeted by the *Tbx4* lung enhancer in our model, we crossed a *Tbx4* lung enhancer–driven rtTA TetO-Cre Dox-inducible line with a Cre-dependent tdTomato reporter line. After Dox administration from P0 to P5, the distal lung of P5 progeny showed tdTomato fluorescence that colocalized with PDGFR-α^pos^/α-SMA^pos^ in myofibroblasts and in pericytes identified by NG2^pos^ membrane staining (Supplemental Figure 4, B and C). tdTomato fluorescence did not colocalize with α-SMA^pos^ smooth muscle cells or with PDGFR-α^pos^ adventitial fibroblasts found in discrete concentric layers in pulmonary artery walls (Supplemental Figure 4A). We corroborated these findings by querying a previously published single-cell RNA-Seq (scRNA-Seq) dataset of WT mouse lung parenchymal cells (47). In the P5 lung, *Tbx4* expression was nearly exclusive to the mesenchymal compartment (99.5% *Tbx4*^pos^ cells, Supplemental Table 1), with myofibroblasts accounting for 61.0% and pericytes for 10.8% of total *Tbx4*^pos^ cells. Few smooth muscle cells and adventitial fibroblasts were *Tbx4*^pos^ at P5 in this lung atlas. Thus, *Tbx4* lung enhancer activity is restricted to specific mesenchymal subpopulations during the saccular stage, as previously reported (48–50).

### Mesenchymal inflammation recruits macrophages to the saccular-stage lung.

**Figure 2:**
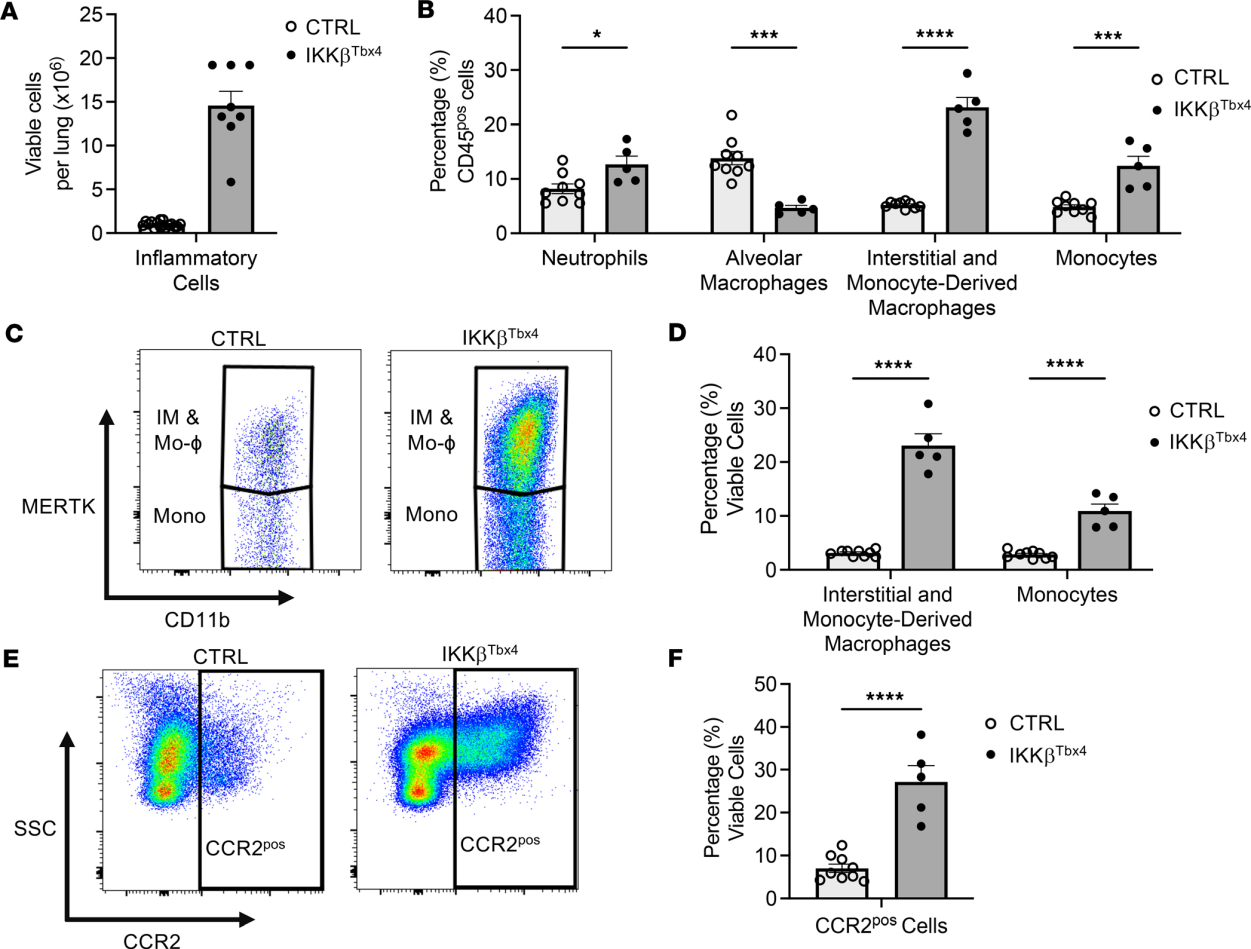
Mesenchymal-derived inflammation recruits macrophages to the saccular-stage lung. Doxycycline was added to drinking water of lactating dams from P0 to P5, and neonatal lungs were harvested on P5. Flow cytometry was used to quantify cells. (**A**) Quantification of viable CD45^pos^ cells in lungs of control (CTRL) and IKKβ^Tbx4^ mice at P5. (**B**) Quantification of myeloid populations in lungs of CTRL and IKKβ^Tbx4^ mice at P5. (**C** and **E**) Flow cytometry plots demonstrating interstitial macrophages and monocyte-derived macrophages (IM & Mo-ϕ [MERTK^pos^, CD11b^pos^]) and monocytes (Mono [MERTK^neg^, CD11b^pos^]) (**C**) and CCR2^pos^ cells (**E**) in CTRL and IKKβ^Tbx4^ lungs at P5 (SSC, side scatter). (**D** and **F**) Quantification of interstitial and monocyte-derived macrophages (**D**) and CCR2^pos^ cells (**F**) in lungs of CTRL and IKKβ^Tbx4^ mice at P5. Data are expressed as mean ± SEM. *n* = 5 to 9 mice per group (**B**, **D**, and **F**); *P < 0.05, ***P < 0.001, and ****P < 0.0001 by unpaired 2-tailed *t* test (**A**, **B**, **D**, and **F**).

We hypothesized that the hypercellular interstitium of P5 IKKβ^Tbx4^ lungs reflected inflammatory cell recruitment. To evaluate this hypothesis, we used flow cytometry to quantify myeloid cell populations in IKKβ^Tbx4^ and littermate control lungs (Supplemental Figure 5A). IKKβ^Tbx4^ lungs had a nearly 10-fold increase in CD45^pos^ inflammatory cells compared with controls at P5 (Figure 2A). Notably, recruitment of circulating monocytes increased the CD11b^pos^/MERTK^pos^ macrophage population in IKKβ^Tbx4^ lungs, consistent with interstitial and monocyte-derived macrophages (Figure 2, B–D). Many recruited cells expressed CCR2, a chemokine receptor expressed on BM-derived monocytes and macrophages (Figure 2, E and F) (51–53). Recruited macrophages primarily localized to the lung interstitium of IKKβ^Tbx4^ lungs (Supplemental Figure 5B).

### Saccular-stage fibroblasts produce chemokines that recruit macrophages.

**Figure 3:**
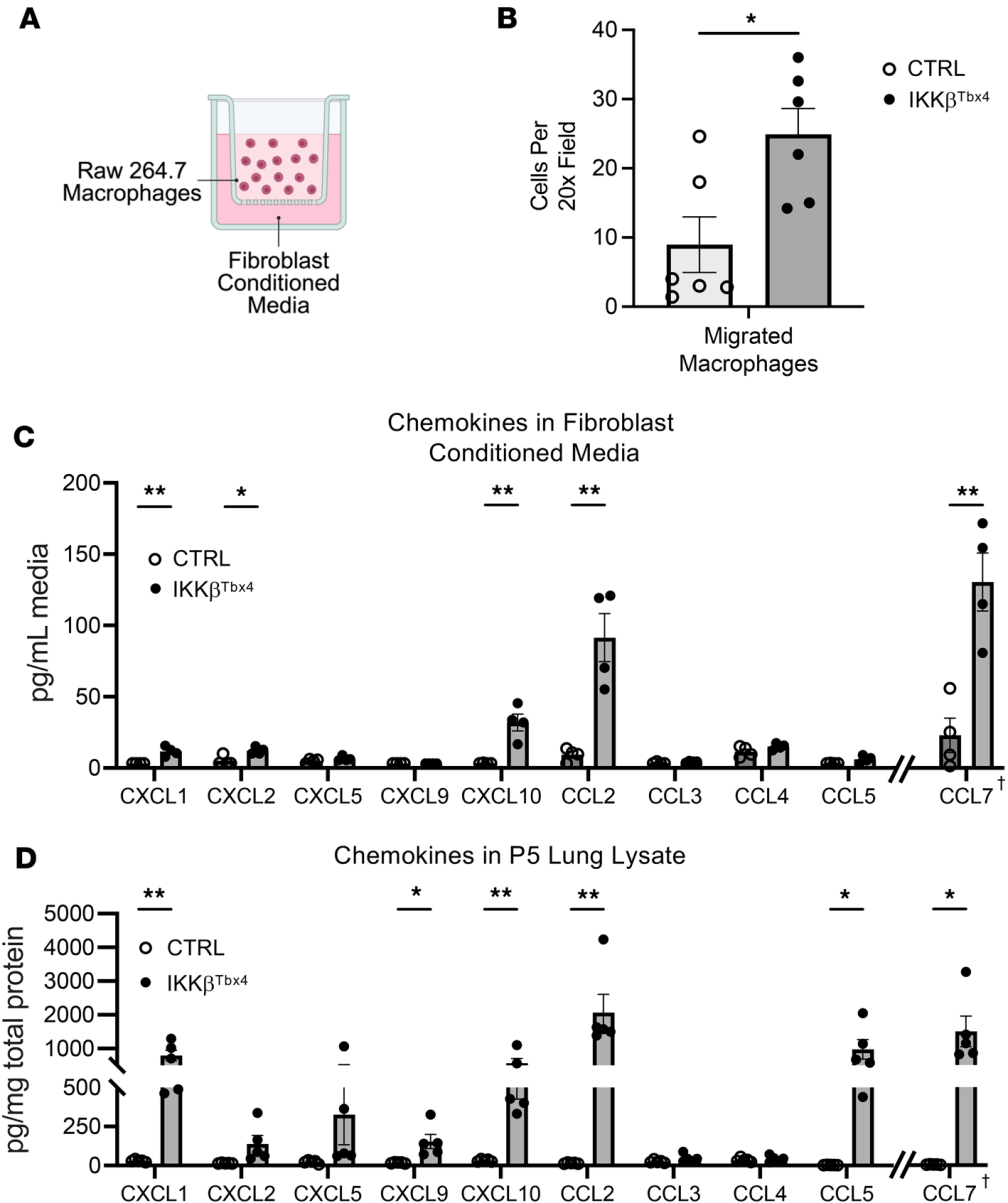
IKKβ^Tbx4^ mouse lung fibroblasts produce chemokines that recruit macrophages. (**A** and **B**) Primary lung fibroblasts were isolated from IKKβ^Tbx4^ and littermate control (CTRL) lungs on P2 and were treated with doxycycline (3 μg/mL in media) at passage 4 or 5. Conditioned media was collected after 24 hours. Conditioned media from CTRL and IKKβ^Tbx4^ lung fibroblasts was added to the bottom well of a modified Boyden chamber. RAW 264.7 cells (50,000 per insert) were added to the top chamber (**A**) and migrated macrophages were fixed, stained, imaged, and counted (**B**). (**C**) Chemokine protein levels were quantified in fibroblast conditioned media using a 32-cytokine/chemokine multiplex assay and CCL7 ELISA. (**D**) Doxycycline was added to drinking water of lactating dams from P0 to P5. Lungs from CTRL and IKKβ^Tbx4^ mice were harvested on P5. Chemokine protein levels were quantified in lung lysate using a 32-cytokine/chemokine multiplex assay and CCL7 ELISA. Full multiplex and ELISA results are shown in Supplemental Tables 2 and 3. Inflammatory protein concentrations in lung lysate were normalized to total lung protein content. Data are expressed as mean ± SEM. *n* = 6 Transwells per group and *n* = 5 fields per Transwell membrane (**B**); *n* = 4 biological replicates per group (**C**); *n* = 5 mice per group (**D**); † = analyte measured using ELISA; *P < 0.05, **P < 0.01, ***P < 0.001, and ****P < 0.0001 by unpaired 2-tailed *t* test (**B**–**D**).

We postulated that mesenchymal chemokine production contributed to monocyte and macrophage recruitment to lungs of IKKβ^Tbx4^ mice. To test this, we generated primary lung fibroblast cultures from IKKβ^Tbx4^ and littermate control mice at P2. After in vitro transgene induction in these cells (Dox 3 μg/mL for 24 hours in serum-free conditions), fibroblast conditioned media was collected for use in macrophage chemotaxis assays using a modified Boyden chamber (Figure 3A). Conditioned media from IKKβ^Tbx4^ fibroblasts increased macrophage chemotaxis in comparison to media from control fibroblasts (Figure 3B), indicating that inflammatory signaling in lung mesenchymal cells released paracrine factors facilitating the recruitment of monocyte-derived macrophages to the saccular-stage lung. Of note, IKKβ activation in primary lung fibroblasts did not induce expression of markers of myofibroblast differentiation (Supplemental Figure 6A).

To determine whether specific chemokines mediated macrophage recruitment, we next quantified chemokine/cytokine expression in fibroblast-conditioned media. Among the cytokines and chemokines that were increased in conditioned media from IKKβ^Tbx4^ fibroblasts (Supplemental Table 2), we measured high levels of CCL2 and CCL7, both ligands of CCR2 (Figure 3C). Lung tissue lysates from transgenic IKKβ^Tbx4^ mice contained higher concentrations of CCL2 and CCL7 compared with controls (Figure 3D, Supplemental Figure 6B, and Supplemental Table 3), and expression of *Ccl2* and *Ccl7* transcripts was also upregulated in IKKβ^Tbx4^ lungs. By comparison, mRNA expression of other known murine CCR2 ligands, *Ccl8* and *Ccl12* (54), was not increased (Supplemental Figure 6C). Together, these data suggest that select CCR2 chemokines produced by the IKKβ^Tbx4^ lung mesenchyme mediate monocyte and macrophage recruitment into the saccular-stage lung.

### Inhibiting recruitment of CCR2^pos^ monocytes prevents structural abnormalities in IKKβ^Tbx4^ lungs.

**Figure 4:**
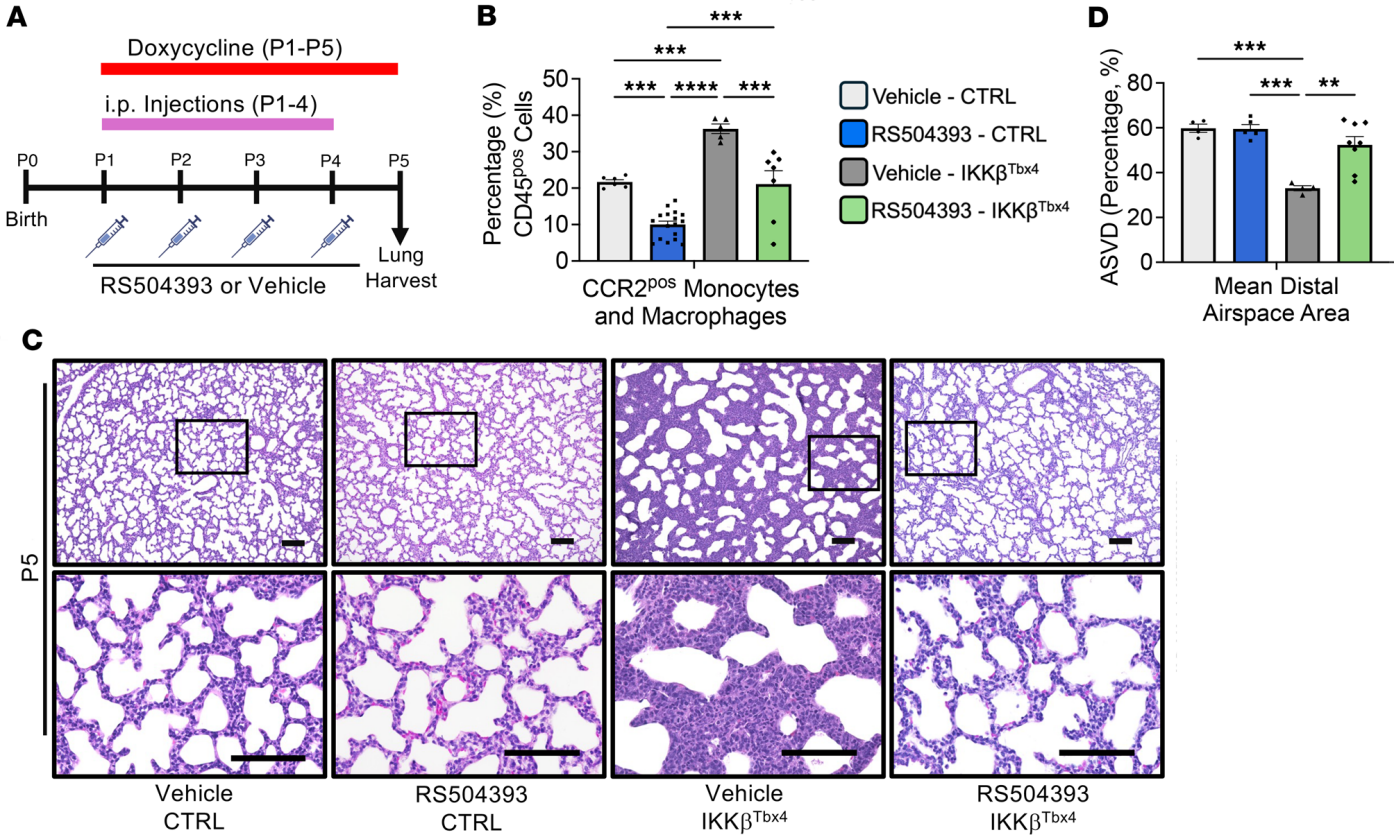
Inhibiting recruitment of CCR2^pos^ monocyte-derived macrophages rescues structural abnormalities in IKKβ^Tbx4^ lungs. (**A**) Schematic of experimental time points. For these experiments, doxycycline was added to drinking water of lactating dams from P1 to P5, and i.p. injections of either vehicle (DMSO, 20 μL) or CCR2 antagonist (RS504393, 2 μg/g) were administered daily from P1 to P4. Lungs were harvested on P5. (**B**) Flow cytometry was used to quantify viable CCR2^pos^ monocytes and macrophages in lungs of vehicle- or RS504393-treated littermate control (CTRL) and IKKβ^Tbx4^ mice at P5. (**C**) Representative H&E-stained lung sections from vehicle- and RS504393-treated mice at P5. (**D**) Morphometric quantification of airspace volume density (ASVD) of vehicle- and RS504393-treated mouse lungs at P5. Data are expressed as mean ± SEM. *n* = 10 to 12 nonoverlapping lung fields per lung (biological replicate) (**D**), *n* = 5 to 17 mice per group (**B**); *n* = 4 to 8 mice per group (**D**); scale bar: 100 μm (**C**); *P < 0.05, **P < 0.01, ***P < 0.001, and ****P < 0.0001 by 1-way ANOVA with Tukey’s multiple-comparison test (**B** and **D**).

Having observed that CCR2^pos^ cells were increased in transgenic lungs, we next tested whether blocking recruitment of CCR2^pos^ monocytes to IKKβ^Tbx4^ lungs would inhibit the observed structural abnormalities. For these experiments, from P1 to P4 we administered i.p. injections daily of vehicle (DMSO) or the small-molecule CCR2 antagonist RS504393 to block recruitment of circulating CCR2^pos^ monocytes prior to lung harvest at P5 (Figure 4A). RS504393 administration reduced CCR2^pos^ monocytes and macrophages in IKKβ^Tbx4^ lungs and rescued the morphometric abnormalities (Figure 4, B–D). Although vehicle-treated IKKβ^Tbx4^ mouse lungs demonstrated interstitial thickening and reduced distal airspace area, lungs of RS504393-treated IKKβ^Tbx4^ mice were indistinguishable from littermate controls. These findings demonstrate that CCR2-mediated monocyte recruitment drives the abnormal lung phenotype in IKKβ^Tbx4^ mice.

### Elastic fiber organization is altered in IKKβ^Tbx4^ lungs.

**Figure 5:**
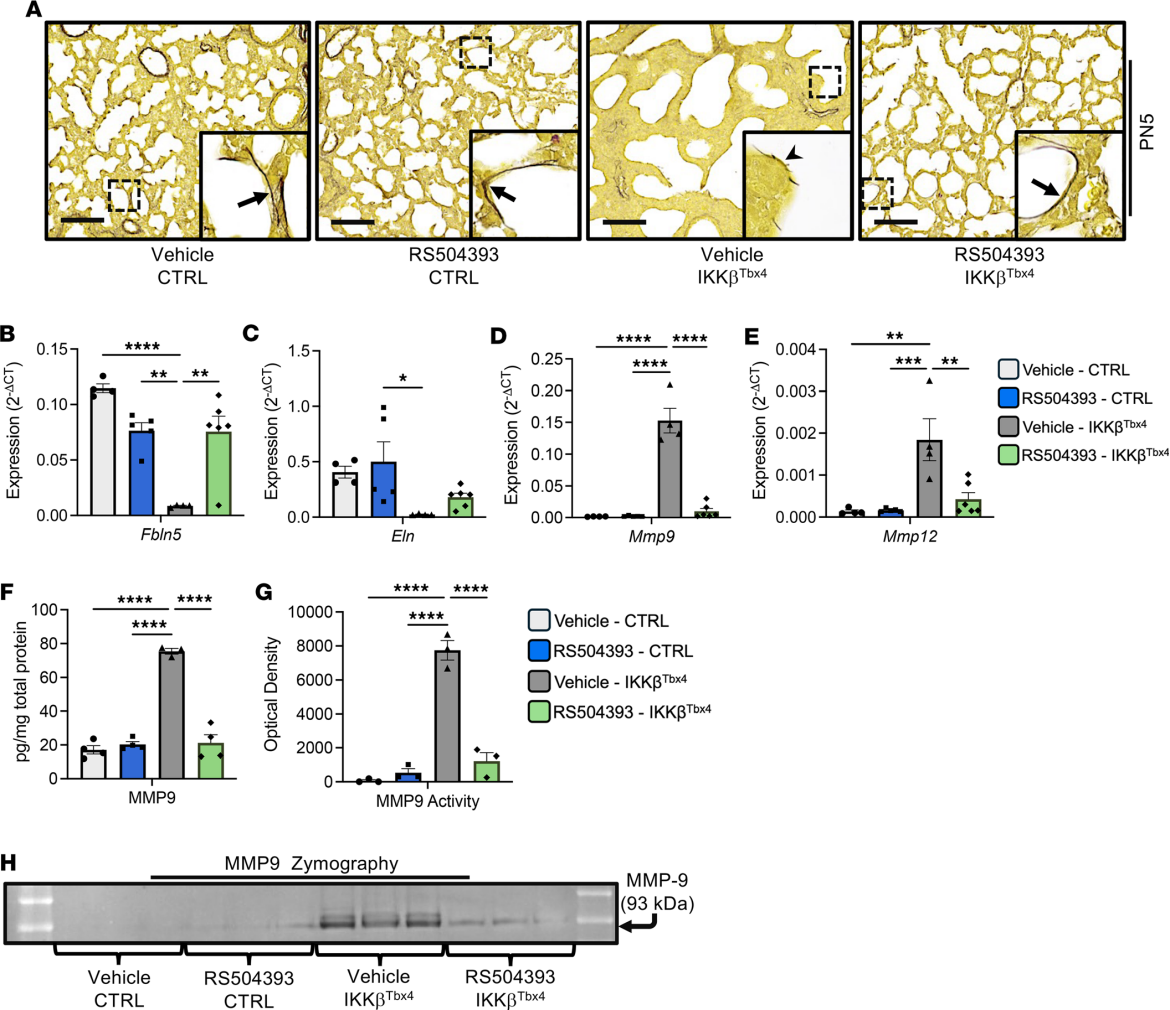
Elastic fiber organization is altered in IKKβ^Tbx4^ lungs and rescued with CCR2 antagonism. Doxycycline was added to drinking water of lactating dams from P1 to P5, and i.p. injections of either vehicle (DMSO, 20 μL) or CCR2 antagonist (RS504393, 2 μg/g) were given daily on P1–P4. Lungs were harvested on P5. (**A**) Representative photomicrographs of Hart’s-stained P5 lung sections from vehicle- or RS504393-treated littermate control (CTRL) and IKKβ^Tbx4^ mice. Arrows denote normal elastic fibers. Arrowheads denote fragmented elastic fibers in vehicle-treated IKKβ^Tbx4^ lungs. Experiments were repeated 4–5 times per group. (**B**–**E**) Gene expression of elastin assembly components (**B** and **C**) and macrophage elastases (**D** and **E**) in the lungs of vehicle- or RS504393-treated CTRL and IKKβ^Tbx4^ mice at P5 measured by qRT-PCR. (**F**–**H**) Lungs from vehicle- and RS504393-treated CTRL and IKKβ^Tbx4^ mice at P5 were homogenized and used to quantify MMP-9 protein levels and enzymatic activity using a mouse MMP-9 ELISA (**F**) and gelatin zymography (**G** and **H**), respectively. Data are expressed as mean ± SEM. *n* = 4 to 6 mice per group (**B**–**E**); *n* = 3 to 4 mice per group (**F**); *n* = 3 mice per group (**H**); scale bar: 100 μm (**A**); *P < 0.05, **P < 0.01, ***P < 0.001, and ****P < 0.0001 by 1-way ANOVA with Tukey’s multiple-comparison test (**B**–**G**).

Precise arrangement of elastic fibers around developing saccules is critical for normal saccular-stage lung development, and loss of this process leads to an emphysema phenotype later in life (55, 56). Because adult IKKβ^Tbx4^ lungs had an emphysematous phenotype with reduced tissue elastance (Figure 1, D–G), we examined elastic fiber deposition in the distal saccular-stage lung. At P5, elastic fibers were organized as cord-like structures around distal airspaces in control lungs, whereas fibers appeared sparse, thin, and discontinuous around airspaces in IKKβ^Tbx4^ lungs (Figure 5A). Abnormalities in elastin deposition persisted into adulthood, and IKKβ^Tbx4^ lungs continued to demonstrate a markedly disorganized elastin network at 2 months of age (Supplemental Figure 7). Importantly, treatment with the CCR2 antagonist RS504393 preserved the structure and organization of elastic fibers in P5 IKKβ^Tbx4^ lungs (Figure 5A).

We next investigated mechanisms by which recruited macrophages could disrupt elastic fiber organization in IKKβ^Tbx4^ lungs. We first investigated components of elastic fiber assembly. Expression of 2 critical components of elastin assembly, *Fbln5* and *Eln*, was downregulated in lungs of vehicle-treated IKKβ^Tbx4^ mice and rescued with CCR2-antagonist treatment (Figure 5, B and C), indicating that recruited CCR2^pos^ macrophages may alter elastic fiber assembly in the saccular-stage lung. Because macrophages produce MMPs that degrade elastic fibers, we also examined whether macrophage-derived MMP-9 or MMP-12 were increased in IKKβ^Tbx4^ lungs. Both *Mmp9* and *Mmp12* were upregulated in lungs of vehicle-treated transgenic mice compared with controls (Figure 5, D and E) and returned to control levels after RS504393 treatment. MMP9 protein levels and enzymatic activity quantified by zymography mirrored the gene expression data and were similarly normalized by RS504393 treatment (Figure 5, F–H). Collectively, these data indicated that monocyte-derived macrophages may disrupt elastin networks through 2 separate mechanisms: by altering elastic fiber assembly or by degrading preassembled elastic fibers through MMP activity.

### Monocyte-derived macrophages disrupt saccular-stage microvascular development.

**Figure 6:**
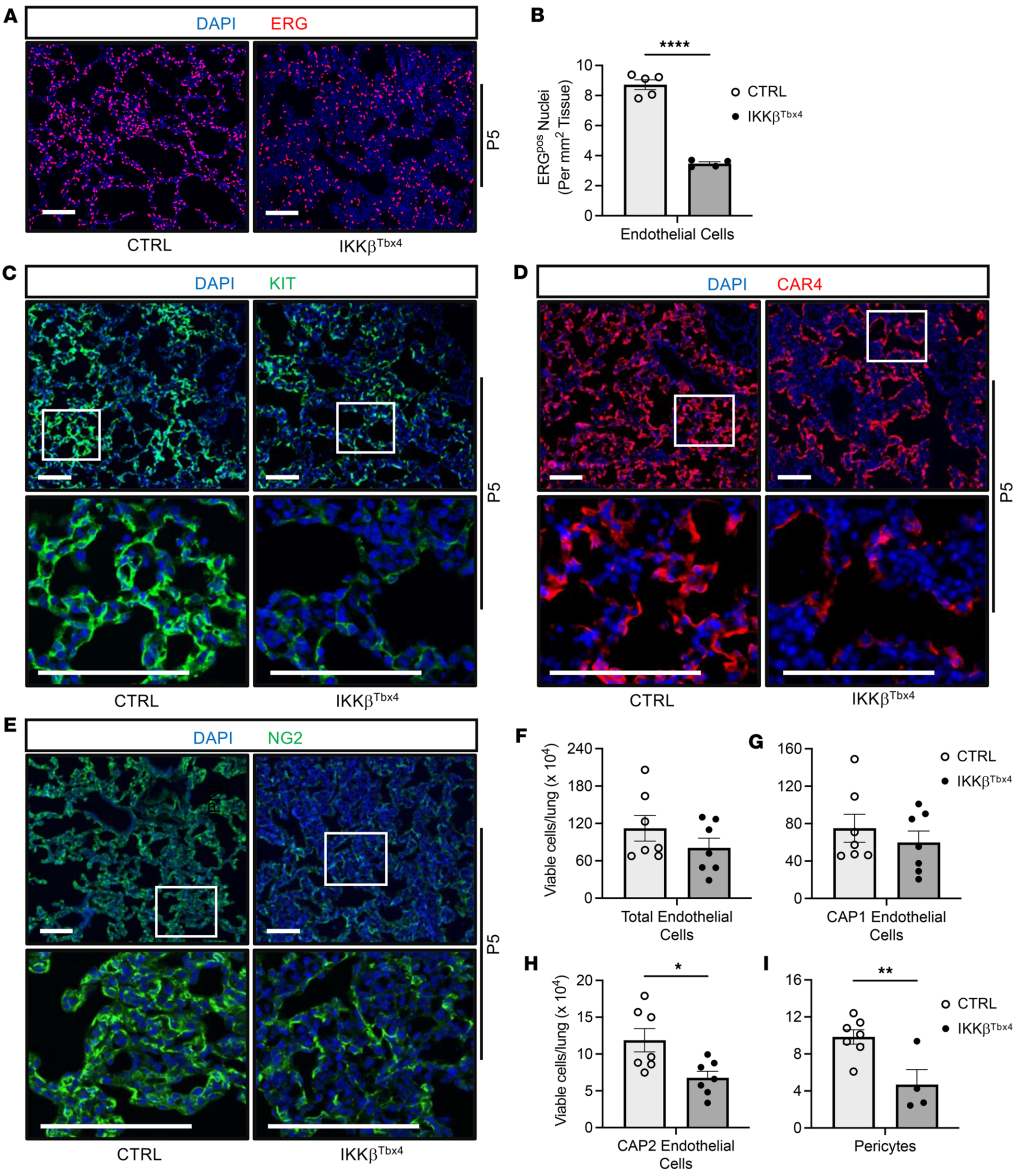
CAP2 endothelial cells and pericytes are reduced in IKKβ^Tbx4^ lungs. Doxycycline was added to drinking water of lactating dams from P0 to P5, and neonatal lungs were harvested on P5. (**A**–**E**) Immunostained lung sections from control (CTRL) and IKKβ^Tbx4^ mice at P5. Immunostaining (**A**) and quantification (**B**) of lung endothelial cell (EC) nuclear marker ERG. Immunostaining for CAP1 EC marker KIT (**C**), CAP2 EC marker CAR4 (**D**), and pericyte marker NG2 (**E**). Nuclei labeled with DAPI (**A**–**E**). Fluorescent imaging was performed using either widefield (**A** and **D**) or confocal microscopy (**C** and **E**). (**F**–**I**) Lung microvascular populations were quantified using flow cytometry. Number of total ECs (**F**), CAP1 ECs (**G**), CAP2 ECs (**H**), and pericytes (**I**) in the P5 lung. Gating strategy to identify capillary subpopulations and pericytes is outlined in Supplemental Figure 9B. Data are expressed as mean ± SEM; 10 to 12 nonoverlapping tissue fields were evaluated per lung (biological replicate), *n* = 4 or 5 mice per group (**B**); *n* = 7 mice per group (**F**–**H**); *n* = 4 to 7 mice per group (**I**); scale bar: 100 μm (**A** and **C**–**E**); *P < 0.05, **P < 0.01, and ****P < 0.0001 by unpaired 2-tailed *t* test (**B** and **F**–**I**).

During the transition to postnatal life, immature capillary endothelial cells differentiate alongside proliferating pericytes to expand a functional microvascular network for gas exchange (47, 57–59). Disrupting vascular patterning during this critical period leads to alveolar simplification (60–64). We therefore examined microvascular development in IKKβ^Tbx4^ mice. Lung endothelial cells were quantified at P5 by immunostaining for the pan-endothelial nuclear marker ERG. IKKβ^Tbx4^ lungs exhibited a marked reduction in endothelial cell numbers compared with littermate controls (Figure 6, A and B). Microvascular organization was also impaired, with the appearance of fewer Endomucin^pos^ vessel structures in transgenic lungs (Supplemental Figure 8A). These vascular abnormalities persisted into adulthood, as IKKβ^Tbx4^ lungs continued to demonstrate reduced microvascular complexity at 2 months of age (Supplemental Figure 8B).

To determine whether microvascular subpopulations contributed to this phenotype, P5 lung sections were immunostained for endothelial subpopulations and capillary-associated pericytes. Staining for both CAP1 (general capillary [gCap]) and CAP2 (alveolar capillary [aCap]) endothelial cell populations was reduced in transgenic lungs, as assessed by KIT and CAR4 staining, respectively (Figure 6, C and D). Similarly, staining for the pericyte markers NG2 and PDGFR-β was decreased in IKKβ^Tbx4^ lungs compared with controls (Figure 6E and Supplemental Figure 9A). Although flow cytometry (Supplemental Figure 9B) did not detect a significant decrease in total PECAM^pos^/Ve-Cadherin^pos^ endothelial or KIT^pos^ CAP1 cells (Figure 6, F and G), both CAR4^pos^/KIT^neg^ CAP2s and PDGFR-β^pos^/CD146^pos^ pericytes were decreased in lungs of IKKβ^Tbx4^ mice (Figure 6, H and I).

**Figure 7:**
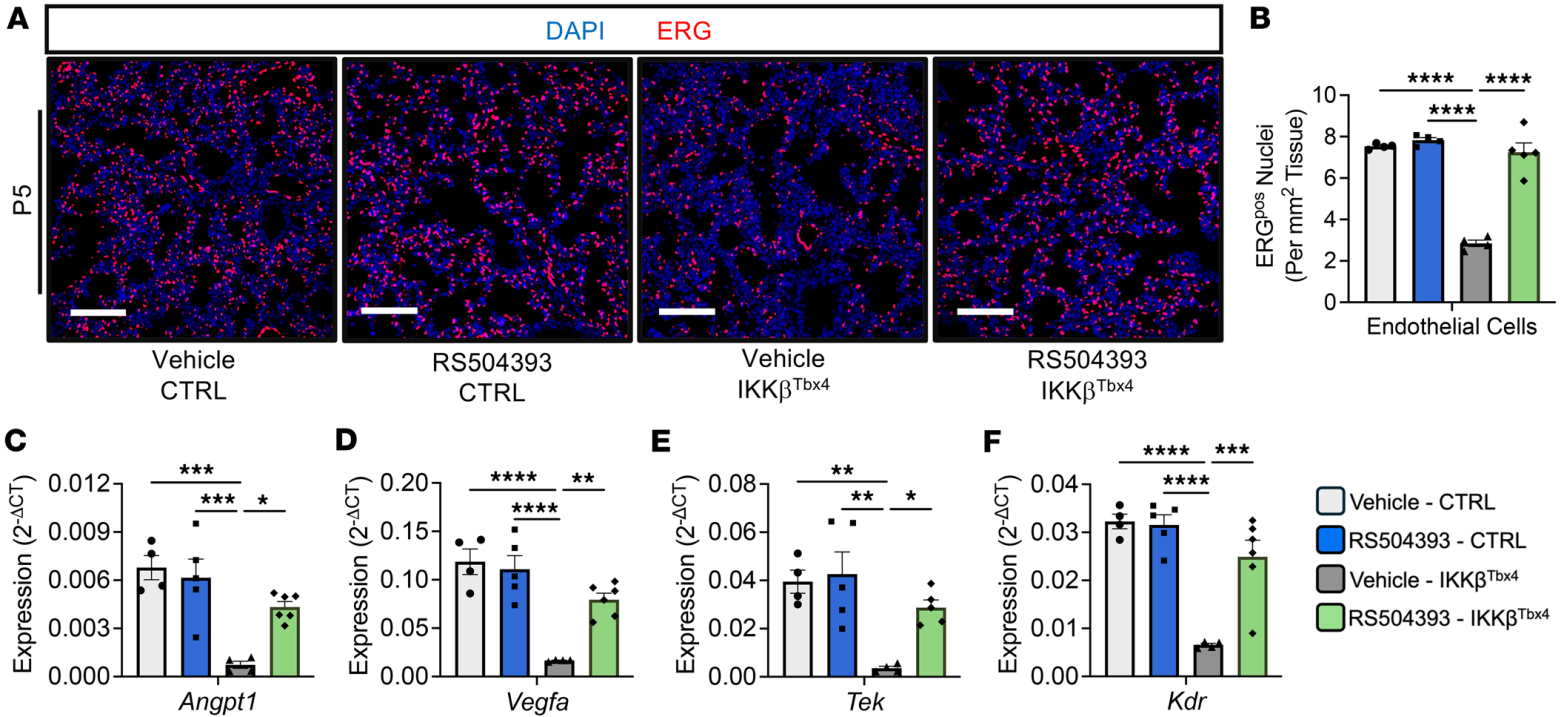
CCR2 antagonism rescues vascular development in IKKβ^Tbx4^ mice. Doxycycline was added to drinking water of lactating dams from P1 to P5, and i.p. injections of either vehicle (20 μL of DMSO) or CCR2 antagonist (RS504393, 2 μg/g) were given daily on P1–P4. Lungs were harvested on P5. (**A** and **B**) Quantification of lung endothelial cells at P5. (**A**) Representative photomicrographs of lung sections from vehicle- and RS504393-treated control (CTRL) and IKKβ^Tbx4^ mice at P5 immunostained for endothelial cell nuclear marker ERG; nuclei are labeled with DAPI. Fluorescent imaging performed using Keyence BZX-800 widefield microscope. (**B**) Quantification of ERG^pos^ nuclei at P5. (**C**–**F**) Gene expression of select proangiogenic growth factors and their cognate receptors in CTRL and IKKβ^Tbx4^ lungs at P5 measured by qRT-PCR. Data are expressed as mean ± SEM; 10 to 12 nonoverlapping tissue fields were evaluated per lung, *n* = 4 or 5 mice per group (**B**); *n* = 4 to 6 mice per group (**C**–**F**); scale bar: 100 μm (**A**); *P < 0.05, **P < 0.01, ***P < 0.001, and ****P < 0.0001 by 1-way ANOVA with Tukey’s multiple-comparison test (**B**–**F**).

CCR2-blockade with RS504393 restored ERG^pos^ endothelial cell numbers in P5 IKKβ^Tbx4^ lungs (Figure 7, A and B). Microvascular architecture was also improved, as demonstrated by Endomucin and CAR4 staining of capillary structures (Supplemental Figure 10, A and B). Given the reduction in CAP2 endothelial cells and pericytes, we examined expression of proangiogenic growth factors associated with these populations. Multiplex analysis of lung lysates revealed reduced levels of VEGF-A in IKKβ^Tbx4^ lungs compared with controls (Supplemental Table 3). Consistent with this, gene expression of *Vegfa*, *Angpt1*, and their cognate receptors *Kdr* and *Tek*, respectively, was decreased in vehicle-treated transgenic lungs and restored with RS504393 treatment (Figure 7, C–F). These data indicate that IKKβ^Tbx4^ mice exhibit abnormal microvascular development during the saccular stage, with disorganized capillary and pericyte architecture and reduced numbers of CAP2 endothelial cells and pericytes. CCR2 inhibition rescued microvascular architecture, endothelial cell numbers, and growth factor expression, indicating that recruitment of CCR2^pos^ cells disrupts signaling required for saccular-stage microvascular maturation.

## Discussion

Despite the considerable morbidity associated with BPD (16, 18, 65), fundamental questions remain about how inflammation alters lung development. Although innate factors that increase the risk of developing BPD (such as gestational age) cannot be modified after birth, the mechanisms driving inflammatory lung injury may still be targetable. Identifying these critical mediators could inform strategies that improve short- and long-term outcomes in neonates at risk of developing BPD. With this goal, we developed the IKKβ^Tbx4^ mouse model, where inflammatory signaling was specifically and transiently activated in the saccular-stage lung mesenchyme. In this model, IKKβ transgene expression was sufficient to cause permanent abnormalities in lung structures, similar to those observed in the BPD lung. Further, mesenchymal inflammation recruited CCR2^pos^ monocyte-derived macrophages, which disrupted the elastic fiber scaffold and impaired microvascular development. Blockade of CCR2-dependent immune cell recruitment rescued lung structure, establishing a mechanistic link between mesenchymal chemokine signaling and structural injury in the saccular-stage lung. The similarities between mouse IKKβ^Tbx4^ and human BPD lungs further support the lung mesenchyme as a potent amplifier of pathological inflammation and reinforce the saccular-stage lung’s vulnerability to inflammatory injury.

IKKβ^Tbx4^ mice recapitulated structural and inflammatory features of human BPD. Histologically, lungs of infants with BPD are characterized by simplified distal airspaces, irregular elastin organization, and dysmorphic capillary architecture (11–15, 66–68), all features demonstrated in the lungs of IKKβ^Tbx4^ mice. Lungs from transgenic mice also demonstrated robust macrophage infiltration, mirroring the increased macrophage burden observed in BPD lungs (10, 68–71). Macrophage-derived inflammation disrupts normal lung development (33, 72), and reducing macrophage recruitment improves alveolarization in animal models of neonatal lung injury (70, 73–77). Similarly, blocking CCR2-dependent macrophage infiltration using RS504393 rescued the lung phenotype of IKKβ^Tbx4^ mice, confirming that monocyte-derived macrophages, recruited by mesenchymal CCL2 and CCL7 production, are the primary drivers of structural injury. Proinflammatory macrophages are increased in human BPD lungs (69, 78, 79), and CCL2 and CCL7 levels are higher in plasma and tracheal aspirates of infants developing BPD (80–82). These parallels to human disease strengthen the evidence that mesenchymal NF-κB–driven inflammation recruits macrophages in BPD and further validate the translational relevance of the IKKβ^Tbx4^ model.

We previously explored the role of epithelial cell–associated inflammation using the IKTA (inhibitory κB kinase β transactivated) mouse model, in which the same activated IKKβ transgene was induced to increase NF-κB activity in the airway epithelium during the saccular stage of lung development (55, 56). Both the IKKβ^Tbx4^ mesenchymal and IKTA epithelial models exhibit a pronounced, but transient, inflammatory response after transgene induction during the saccular stage. However, whereas the IKKβ^Tbx4^ model was associated with robust macrophage recruitment to the lung and interstitial thickening, the IKTA model produced a neutrophil-predominant inflammatory response with dilated, emphysematous-appearing airspaces. Prior reports also indicate that in the developing lung endothelium, NF-κB activity is protective against exogenous inflammatory injury and required for normal vascular development (83–85). Thus, the effects of NF-κB activation are cell-type dependent. Developmental stage also influences the consequences of NF-κB–mediated inflammatory signaling. Constitutive expression of IKKβ in Twist2^pos^ mesenchymal cells (Twist2-IKKβca mice) activated NF-κB signaling and recruited macrophages, yet no changes were observed in airway branching or elastin deposition in the canalicular-stage lung (86). Similarly, in IKTA mice, only saccular (but not alveolar) stage NF-κB activation altered lung development (56). The contrasting phenotypes across these models demonstrate how the context of NF-κB activation determines the downstream immune response and pattern of structural injury. Understanding how the cellular source and timing of NF-κB signaling shape its impact will be important when targeting this pathway using novel therapeutics.

IKKβ^Tbx4^ mice build upon prior evidence that the saccular stage is a period of developmental vulnerability, during which even brief inflammatory exposures can permanently alter lung structure (33, 55, 87). Despite distinct early phenotypes, both IKKβ^Tbx4^ and IKTA mice demonstrated lasting abnormalities in elastin deposition and emphysematous enlargement of the distal airspaces at 2 months of age, mirroring the durable structural changes observed in BPD lungs (56). Although often regarded as a disease of prematurity, BPD has consequences that extend well beyond infancy; affected individuals exhibit persistent reductions in lung function (18, 88–90), and those with severe disease are at risk for chronic obstructive pulmonary disease in adulthood (91–94). Defining how mesenchymal inflammation disrupts saccular-stage development programs may inform early interventions to prevent lifelong morbidity.

Elastin is assembled during a narrow window of lung development and, under normal conditions, undergoes minimal turnover throughout life (95). Thus, disruption during the saccular stage can permanently compromise the elastic fiber scaffold (56). IKKβ^Tbx4^ lungs demonstrated sparse, fragmented elastic fibers lining distal airspaces. Abnormalities in elastin assembly and degradation have been strongly implicated in the pathogenesis of BPD, with disordered elastin organization described in both animal models of neonatal lung inflammation and in preterm infants exposed to saccular-stage inflammation (31, 55, 96–100). Macrophage recruitment to IKKβ^Tbx4^ lungs reduced the expression of critical elastin assembly genes and increased production of macrophage-derived MMPs, disrupting the elastin network through both impaired assembly and enhanced degradation. Importantly, CCR2 inhibition with RS504393 prevented macrophage recruitment and rescued the elastin phenotype, confirming a direct role for recruited macrophages in elastin dysregulation.

Coordinated maturation of the lung microvasculature during the saccular stage is essential for normal alveolarization (60–63, 101, 102). Lungs from infants with BPD exhibit capillary rarefaction, dysmorphic capillary architecture, and aberrant endothelial patterning (11, 12, 68, 103–106). Recent scRNA-Seq studies have identified 2 major pulmonary capillary endothelial subpopulations: proliferative CAP1 cells that support vascular growth and repair, and CAP2 cells that differentiate from CAP1s during the saccular stage to facilitate gas exchange (34, 47, 63, 107, 108). IKKβ^Tbx4^ lungs demonstrated reduced and disorganized microvascular structure with decreased CAP2 and pericyte numbers. Expression of *Vegfa* and *Angpt1*, growth factors critical for CAP2 and pericyte development, was decreased in transgenic lungs. RS504393 rescued expression of both genes and restored ERG^pos^ and CAR4^pos^ endothelial cell staining at P5, demonstrating that CCR2-dependent macrophage recruitment impairs proangiogenic signaling directing saccular-stage microvascular development. In line with these findings, augmenting VEGF-A signaling improves distal lung development across multiple neonatal injury models (60, 101, 109) and is a future direction for our studies using the IKKβ^Tbx4^ model.

Capillary loss and pathological vascular remodeling during lung development can predispose to chronic pulmonary vascular disease (110–113); adult survivors of BPD remain at elevated risk across the lifespan (114). Pulmonary hypertension (PH) is a common comorbidity of BPD (BPD-PH), affecting as many as 40% of infants with severe disease (115). Infants with BPD-PH are at a higher risk of adverse clinical outcomes, demonstrating increased neurodevelopmental impairment, higher rates of tracheostomy placement, and a 5- to 6-fold increase in mortality compared with infants who have only BPD (115–118). Constitutive overexpression of IL-6 or TNF-α in the mouse lung during development is sufficient to cause PH (119, 120), and inhibition of cytokine signaling protects against PH in multiple neonatal injury models (110, 121). Macrophage depletion similarly prevents hyperoxia-induced PH in neonatal rodents (122). IKKβ^Tbx4^ mice exhibit persistent capillary rarefaction and a macrophage-dependent reduction in proangiogenic signaling, making PH a plausible long-term consequence. Experiments to address this are underway.

Recent studies demonstrate that inflammatory injury drives dynamic shifts in capillary endothelial subpopulations, though the specific patterns and timing of these changes remain an area of active investigation (45, 57, 102, 105, 123–125). IKKβ^Tbx4^ lungs exhibited decreased CAP2 and pericyte numbers at P5, but the fate of these populations beyond this early time point has not yet been characterized. Bidirectional signaling between endothelial cells and pericytes is required for normal capillary network formation. The interdependence of these cell populations has been illustrated by studies in which disrupting postnatal pericyte signaling was sufficient to cause alveolar simplification with reduced microvascular complexity (64), and in which primary endothelial dysfunction was shown to reduce pericyte numbers (63). The loss of CAP2 endothelial cells and pericytes in IKKβ^Tbx4^ lungs raises important questions about how mesenchymal-derived inflammation impairs coordinated microvascular development.

Although our data indicate that recruited macrophages mediate the observed vascular abnormalities in IKKβ^Tbx4^ lungs, direct effects of the mesenchymal secretome on vascular development cannot be fully excluded. Additionally, wet-to-dry lung weight ratios did not indicate pulmonary edema in IKKβ^Tbx4^ mice, but this measure reflects total lung water content relative to tissue mass and may have limited sensitivity for detecting changes in vascular permeability (126). Given the reduction in pericytes, which are critical regulators of capillary barrier function, a targeted assessment of vascular integrity will be a focus of future studies.

In summary, we demonstrate that mesenchymal-derived inflammation in the saccular-stage lung causes lasting disruptions of lung structure. Activation of NF-κB in the lung mesenchyme upregulated CCL2 and CCL7, recruiting CCR2^pos^ macrophages. Despite a brief interval of inflammation, IKKβ^Tbx4^ mice exhibited durable impairment of lung development, specifically dysregulated elastic fiber deposition and impaired microvascular development. These findings establish mesenchyme-macrophage crosstalk as a central contributor to BPD pathogenesis. IKKβ^Tbx4^ mice model the core features of human BPD, enabling further investigation of how inflammation disrupts specific developmental programs during the saccular stage. Future work will address how recruited macrophages disrupt vascular development, how the microvasculature remodels after early endothelial and pericyte loss, and the impact of these features on adult cardiopulmonary disease.

## Methods

### Sex as a biological variable.

Animals of both sexes were included in the analysis for all experiments completed at P5 and 2 months of age. No discernible sex differences were noted in the analysis.

### Reagents.

Immunostaining was completed using anti-Endomucin (ab106100), anti-α smooth muscle actin (ab5694), and anti-ERG (ab92513) from Abcam; anti-phospho-NF-κB p65 (Cell Signaling Technology, 3033); anti-CD117/c-Kit (KIT, AF1356), anti-carbonic anhydrase IV/CA4 (CAR4, AF2414), and anti-PDGFR-β (AF1042) from R&D Systems; and anti-NG2 (AB5320) from MilliporeSigma. All secondary antibodies used in immunofluorescent staining were highly cross-absorbed donkey IgG conjugated to Alexa Fluor Plus (Invitrogen) fluorophores. Autofluorescence was minimized in paraffin-embedded lung sections using TrueBlack Plus Autofluorescence Quencher (Biotium, 23014-T). Nuclei were stained with DAPI solution (Thermo Fisher Scientific, 62248). Coverslips were mounted using ProLong Gold Antifade reagent (Life Science Technologies, P36930).

The primary antibodies used for flow cytometry and FACS are listed in Supplemental Table 4. Otherwise, donkey anti-goat PE-conjugated cross-absorbed F(ab’)^2^ (Invitrogen, 31860) was used as a secondary antibody for anti-CAR4. DAPI (Thermo Fisher Scientific, 62248) labeling was used to assess cell viability in all flow experiments.

### Mouse model design.

All mice used in this study were maintained on a C57BL/6J background. WT C57BL/6J mice used for breeding were obtained from The Jackson Laboratory (strain 000664). To selectively induce expression of IKKβ and activate NF-κB in mesenchymal cells in the saccular-stage lung, we crossed hemizygous mice containing a FLAG-tagged form of activated human IKKβ under the control of the tetracycline operator-minimal CMV promoter (IKKβ mice) (127) with heterozygous mice expressing reverse tetracycline transactivator (rtTA) under the control of the 5.5 kb mouse *Tbx4* lung enhancer (Tbx4-rtTA mice) (48). In the saccular-stage mouse, *Tbx4* is primarily expressed in lung mesenchyme (48–50). Approximately 25% of mice from the above-described matings expressed both the IKKβ and Tbx4-rtTA transgenes; we designated these mice IKKβ^Tbx4^. The remaining littermate mice, which carried only IKKβ, only Tbx4-rtTA, or neither transgene, were used as controls. To induce transgene expression in neonatal lungs, lactating dams were administered Dox (Sigma-Aldrich, D9891) in drinking water (2 g/L of water) during the saccular stage from P0 to P5 (or from P1 to P5; see *Monocyte depletion* below). Lungs were then harvested at P5 or 2 months of age.

To assess cellular transgene expression patterns under the control of the *Tbx4* lung enhancer, we crossed Tbx4-rtTA/TetO-Cre mice with a tdTomato reporter mouse (B6.Cg-Gt(ROSA)26Sor^tm14(CAG-tdTomato)Hze^/J, [The Jackson Laboratory, JAX strain 007914]). Upon Dox administration, progeny from these crossings (referred to as Tbx4^tdTomato^ mice), expressed cytosolic tdTomato fluorescence in cells expressing the *Tbx4* lung enhancer. For fluorescent labeling of *Tbx4*^pos^ cells in Tbx4^tdTomato^ neonatal lungs (as with IKKβ^Tbx4^ mice), lactating dams were administered Dox (Sigma-Aldrich) in drinking water (2 g/L of water) from P0 to P5 and harvested at P5.

### Monocyte depletion.

To deplete recruited CCR2^pos^ cells, neonatal mice received i.p. injections of a small-molecule CCR2 antagonist RS504393 (2 μg/g body weight) from Sigma-Aldrich (SML0711) or vehicle DMSO (20 μL, 28% DMSO in PBS) from Sigma-Aldrich daily from P1 to P4 (128, 129). For these experiments, Dox administration was delayed by 1 day (administered from P1 to P5) to align with injections for monocyte depletion. Lungs were then harvested at P5.

### scRNA-Seq analysis.

To determine the cellular expression of *Tbx4* in the P5 mouse lung, we reexamined a previously published scRNA-Seq dataset of CD45- and Ter119-depleted WT mouse lung parenchymal cells using a web-based explorer (https://lungcells.app.vumc.org/) (47). Cells were previously sequenced using the 10x Genomics Chromium platform. For analysis, we queried *Tbx4* expression among previously annotated cell populations.

### Lung histology.

For paraffin-embedded samples, lungs of P5 mice were perfused with PBS, isolated, and fixed for 24 hours in 10% neutral buffered formalin before automated tissue processing (Leica) and paraffin embedding using standard methods. Lungs from mice at 2 months of age (8–10 weeks) were inflation-fixed with 10% formalin (25 cm H_2_O inflation pressure) before harvest. Paraffin-embedded lung tissue was sequentially sectioned using a rotary microtome at 5 μm thickness and adhered to Superfrost Plus microscope slides (Thermo Fisher Scientific) for further processing. H&E staining was completed using paraffin-embedded tissue sections for assessment of lung morphology using standard methods before imaging using brightfield microscopy with a Keyence BZX-810 inverted widefield microscope (55, 56).

For frozen sections, lungs of P5 mice were perfused with PBS, isolated, and fixed in 4% paraformaldehyde at 4°C overnight before embedding and freezing in Tissue-Tek OCT media (Sakura). In samples expressing endogenous fluorescent protein, fixation was limited to 30 minutes at room temperature. Frozen tissue blocks were sequentially sectioned at –20°C using a manual cryostat at 8 μm thickness, plated on Superfrost Plus microscope slides, and allowed to adhere for 30 minutes at room temperature before storage at –80°C for future immunostaining.

### Lung morphometry.

To conduct morphometric analysis of H&E-stained lung sections from neonatal and adult mice, 10 to 12 nonoverlapping lung fields were imaged across multiple lobes within a single lung section from each mouse. Five to 9 mice were imaged per group. Tiff images of H&E-stained sections were exported and analyzed using Fiji (130) software (version 2.16.0/1.54p) by a trained investigator blinded to animal genotype and treatment status, as previously described (55, 56). Analysis was performed with the semiautomated tool AlveolEye as previously described (131). To determine the proportion of distal airspace area in P5 neonatal lung sections, airspace volume density of distal lung saccules was measured using images obtained at 40× original magnification. Mean linear intercept was measured using 3 test lines overlapped on images taken at 20× original magnification from mice at 2 months of age**.** With the assistance of structural screening by AlveolEye, large-caliber airspaces and large pulmonary vessels were manually identified and excluded from the distal airspace before measurements and analysis.

### Lung wet-to-dry weight ratios.

To determine the lung wet-to-dry weight ratio as a surrogate measure for pulmonary edema (132), P5 mouse lungs were perfused with PBS and isolated en bloc. Whole lung was weighed immediately after harvest and again after 16 hours of oven dehydration at 65°C (133).

### Immunofluorescent staining and ERG quantification.

Immunofluorescent staining was performed using both paraffin-embedded and frozen lung tissue sections. To prepare paraffin-embedded lung tissue for immunostaining, sections were rehydrated using standard techniques, subjected to antigen retrieval with pH 6.5 citrate buffer (Sigma-Aldrich, C9999), and blocked for 10 minutes at room temperature using Power Block per the manufacturer’s recommendations (Biogenex Labs, HK0855K). For frozen lung tissue immunostaining, sections were brought to room temperature, washed in PBS to remove excess OCT, permeabilized for 10 minutes using 0.1% Triton X-100 (Sigma-Aldrich), and blocked for 30 minutes with 1% BSA and 5% horse serum in PBS. After initial steps, all lung sections were incubated with primary antibody in PBS-Tween (0.05%) overnight at 4°C. Sections were subsequently stained with secondary antibody in PBS-Tween (0.05%) for 1 hour at room temperature and briefly incubated with DAPI nuclear stain. Before cover-slipping, paraffin-embedded lung sections were treated with TrueBlack Plus Autofluorescence Quencher per the manufacturer’s recommendations. All slides were mounted with ProLong Gold Antifade Mountant and allowed to cure for at least 24 hours before imaging. Imaging of ERG, Endomucin, and CAR4 staining was performed using a Keyence BZX-810 inverted fluorescent widefield microscope. Other fluorescent images were taken on a Nikon TiE inverted spinning disk confocal microscope equipped with a Yokogawa X1 spinning disk head and Photometrics Prime 95B camera.

To quantify endothelial cells in P5 lungs, lung sections were immunostained for endothelial nuclear marker ERG and imaged with a Keyence BZX-810 inverted fluorescent widefield microscope at 10× and 40× original magnification. Ten to 12 nonoverlapping high-power lung fields were imaged across multiple lobes within a single lung section from each mouse. Four to 5 mice were included per group. Tiff images of ERG-stained sections were exported, and ERG^pos^ nuclei were quantified using Fiji software. Specifically, a threshold was uniformly applied to all ERG-fluorescent images to identify regions of positive staining; ERG^pos^ cells were counted using the Analyze Particles function. The number of ERG^pos^ cells was normalized to the DAPI-fluorescent tissue area (mm^2^) of each image. ERG fluorescence localized outside regions of DAPI staining was considered nonspecific and excluded from analysis. ERG^pos^ nuclei per tissue area was averaged for each animal.

### Hart’s elastic fiber staining.

Elastic fibers were visualized using Hart’s elastin staining as previously described (55). Briefly, paraffin-embedded lung sections were rehydrated and incubated with resorcin-fuchsin (Electron Microscopy Sciences) overnight to stain elastic fibers. Tartrazine in saturated picric acid (Electron Microscopy Sciences) was used to counterstain elastin-stained sections. Slides were imaged using brightfield microscopy with a Keyence BZX-810 inverted widefield microscope.

### Mouse lung fibroblast cultures and generation of conditioned media.

Neonatal mouse lung fibroblasts were isolated using previously described methods (55). Briefly, neonatal mouse lungs at P2 were perfused with sterile PBS and harvested. Lungs were minced with scissors and enzymatically digested. The resulting cell suspension was centrifuged, resuspended in fresh medium, and sequentially filtered. Filtrate from the third filtration was plated on 60 mm cell culture dishes in DMEM containing 10% FBS overnight and passaged or cryopreserved for use in later experiments. All experiments utilizing fibroblast cultures were completed using cells from the fourth or fifth passage. For in vitro experiments, 3 × 10^5^ IKKβ^Tbx4^ or control lung fibroblasts were cultured on 6-well plastic dishes and treated with Dox (3 μg/mL) for 24 hours prior to harvest. The supernatant containing conditioned media was collected and centrifuged (500*g*) to pellet cellular debris. The remaining fibroblasts were harvested in TRIzol for later isolation of total RNA. Both components were frozen at –80°C for future use.

### Mouse lung macrophage cultures.

RAW 264.7 macrophages (ATCC, TIB-71) were cultured in ATCC-formulated DMEM (ATCC, 30-2002) with 10% FBS. Macrophage cultures were passaged (1:6) every 2–3 days, when cells reached 70%–90% confluency.

### Macrophage migration assay.

Experiments utilized a modified Boyden chamber with Transwell filters containing 5 μm pores (Corning, CLS3421) placed in a well of a 12-well tissue culture plate as previously described (134). RAW 264.7 macrophages (50,000 per insert) were placed on top of the Transwell filter in 0.1 mL serum-free DMEM. Conditioned media (0.4 mL) was collected (as above) from IKKβ^Tbx4^ and littermate control fibroblast cultures and added to the bottom well. After a 4-hour incubation at 37°C, macrophages still present on the top of the filter were removed by clearing with a sterile cotton swab. Migrated macrophages on the underside of the Transwell membrane were fixed in 4% paraformaldehyde and stained using the Hema 3 Manual Staining System (Thermo Fisher Scientific, 23-123869) per the manufacturer’s instructions. Five nonoverlapping 20× images of the membrane were taken using a brightfield microscope, and the number of migrated cells per field was quantified.

### Lung function measurements.

After transgene induction with Dox from P0 to P5, lung function was quantified by flexiVent apparatus (SCIREQ) in IKKβ^Tbx4^ and littermate control mice at 2 months of age using previously established methods (135, 136). Briefly, mice were anesthetized using pentobarbital sodium (85 mg/kg) before an 18-gauge tracheostomy tube was placed in the trachea. Mice were mechanically ventilated using the SCIREQ flexiVent apparatus at 150 breaths/min and a 10 mL/kg tidal volume before obtaining lung function measurements. Respiratory system elastance and compliance were assessed using the flexiVent Snapshot model. Tissue elastance was determined using a constant phase model, which distinguishes between airway and tissue mechanics. FEV0.1 (volume expired over initial 0.1 seconds of exhalation) was obtained using a forced expired volume maneuver, which mimics clinical spirometry measurements.

### Flow cytometry and FACS.

Flow cytometry to quantify myeloid cells was completed using previously described methods (56). Briefly, mouse lungs at P5 were perfused with PBS and harvested. Lungs were minced and enzymatically digested for 20 minutes at 37°C using collagenase XI (0.7 mg/mL; Sigma-Aldrich, C7657), type IV DNase (30 μg/mL; Sigma-Aldrich, D5025), and Dispase (0.25 mg/mL; Gibco, 17105-041). Digested cells were filtered through a 70 μm filter to obtain a single-cell suspension and RBCs were lysed using ACK lysing buffer (Gibco) for 3 minutes at room temperature before halting the reaction with cold PBS. Cells were labeled with conjugated primary antibodies and analyzed using a 5-laser LSRFortessa analytical flow cytometer and FlowJo software version 10.10.0 (both from BD Biosciences). DAPI was used to determine cell viability. The total number of cells per lung was determined by preparing a single-cell suspension (as above) and counting using a TC20 Automated Cell Counter per the manufacturer’s instructions (Bio-Rad).

To isolate individual populations of lung parenchyma, transgenic mouse lungs harvested at P4 were processed for flow cytometry as described above. Cells were sorted using FACS with a 5-Laser FACS Aria III (BD Biosciences) cell sorter into epithelial (CD45^neg^, EpCAM^pos^, CD31^neg^), mesenchymal (CD45^neg^, EpCAM^neg^, and CD31^neg^), and endothelial (CD45^neg^, EpCAM^neg^, CD31^pos^) cell populations. Sorted cells were pelleted and resuspended in TRIzol reagent (Invitrogen) before freezing at –80°C for future RNA extraction.

Flow cytometry was also used to quantify lung microvascular subpopulations. Samples were prepared as above with the following protocol modifications to enhance yield and viability (57, 137). Mouse lungs were harvested and minced as above, but were digested with Liberase TM (Roche, 540119001) and type IV DNase (100 μg/mL) for 30 minutes at 37°C before quenching with 10% FBS in PBS. To enhance antibody specificity, cells were blocked with CD-32 Fc block (BD Biosciences, 553141) according to the manufacturer’s recommendations. A subset of samples was stained with a fluorescent-conjugated secondary antibody when required. Finally, for precise quantification of cell populations, Precision Counting Beads (BioLegend, 424902) were added to stained single-cell suspensions immediately before analysis, which was completed with a 3-laser LSRFortessa analytical flow cytometer (BD Biosciences). Cells and counting beads were analyzed using FlowJo software, and lung cell populations were quantified per the manufacturer’s instructions.

### RT-qPCR.

Total RNA was isolated from whole neonatal lungs, cultured lung fibroblasts, or FACS-sorted lung parenchymal cells with TRIzol reagent (Invitrogen) using standard procedures. First-strand cDNA was synthesized using oligo-dT primers and the Superscript VILO cDNA Reverse Transcriptase kit, which uses a Moloney murine leukemia virus (MMLV) reverse transcriptase (Invitrogen, 11754050). PCR primers were designed using the PrimerQuest design tool (Integrated DNA Technologies). Target gene forward (sense) and reverse (antisense) primer sequences are listed in Supplemental Table 5. Two-step real-time PCR was performed using an IQ5 thermocycler and SYBR Green detection system (Bio-Rad). Target gene expression was normalized to housekeeping gene *Actb* in each sample. Fold-change in gene expression was calculated using the 2^–ΔΔCt^ method (138). Gel electrophoresis was used to verify the size of each amplified gene product.

### Protein quantification using ELISA and multiplex assay.

Tissue lysates for protein analysis were generated by sonicating the right-upper lung lobe in 0.5× RIPA (Pierce, 89900) diluted in PBS containing protease and phosphatase inhibitor cocktails (Roche, 04-693-132-001 and 04-906-845-001). Samples were centrifuged, supernatant was collected, and total protein was quantified using a bicinchoninic acid protein assay kit (Pierce, 23225). Conditioned media was prepared as previously described (see *Mouse lung fibroblast cultures and generation of conditioned media*), and total protein was also quantified using a bicinchoninic acid protein assay kit.

Cytokines/chemokines in whole lung tissue lysates and fibroblast conditioned media were quantified using a magnetic bead 32-multiplex assay (MilliporeSigma, MCYTMAG-70K-PX32) in duplicate per the manufacturer’s instructions. The multiplex assay was performed on the Luminex MAGPIX platform in the Vanderbilt Hormone and Analytical Services Core. Mouse CCL7 (Abcam, ab205571) and MMP-9 ELISAs (Abcam, ab253227) were used to quantify CCL7 and MMP-9 expression per the manufacturer’s recommended methods. Cytokine concentrations were normalized to total protein content of the sample where indicated.

### Gelatin zymography.

Lung lysates were prepared as described above. First, 20 μg protein per sample was loaded onto an acrylamide gel containing 0.1% gelatin and subjected to electrophoresis. Gels were prepared and stained with Coomassie blue using standard methods (139). After destaining, zymograms were imaged, and MMP-9 bands (93 kDa) were quantified by densitometry analysis with Fiji software using standard methods (130).

### Statistics.

Pairwise comparisons were done using an unpaired 2-tailed Student’s *t* test or a 1-way ANOVA with Tukey’s correction for multiple comparisons. All analyses were performed using GraphPad Prism version 10.6.0. Data are shown as mean ± SEM unless otherwise noted. A *P* value less than 0.05 was used to determine statistical significance.

### Study approval.

The experiments in this study were conducted in accordance with the guidelines outlined by the US Public Health Service Policy on the Humane Care and Use of Laboratory Animals. All animal experimental protocols were approved by the IACUC at Vanderbilt University Medical Center (protocol M1800030).

### Data availability.

All reagents used in this study will be made available upon reasonable request to the corresponding author. Values for all data points in the graphs are provided in the Supporting Data Values file.

## Author contributions

BCC, JCL, BCE, SD, RVDM, WH, ALS, CS, LP, EJP, DCN, BWR, SHG, and JTB designed and performed experiments and analyzed data. BCC, JCL, CS, LP, DSN, HT, and JTB performed histological analysis. WS, EJP, LSP, and BWR provided methodological support. WS provided the Tbx4-rtTA mouse line. BCC, BWR, TSB, SHG, and JTB conceived of and designed the experiments. BCC and JTB drafted the original manuscript. BCC, BWR, LSP, TSB, SHG, and JTB revised the manuscript. All authors reviewed and approved the final manuscript.

## Conflict of interest

The authors have declared that no conflict of interest exists.

## Funding support

This work is the result of NIH funding, in whole or in part, and is subject to the NIH Public Access Policy. Through acceptance of this federal funding, the NIH has been given a right to make the work publicly available in PubMed Central.

- NIH grants HL094296, HL105334, and HD087023 (supporting BCC as a Research Scholar).
- NIH grants HL122554 and HL136664 (to DCN).
- NIH grant HL163195 (to EJP).
- NIH grant HL150617 (to SHG).
- NIH grant HL157373 (to JTB).
- Department of Veterans Affairs grant IK2BX003841 (to BWR).
- SHG is the Julia Carell Stadler Professor of Pediatrics at the Monroe Carell Jr. Children’s Hospital.

## Supplementary Material

Supplemental data

Unedited blot and gel images

Supporting data values

## Version 1 06/02/2026

In-Press Preview

## Version 2 07/22/2026

Electronic publication
