# Supplementary material for: Mesenchyme-derived inflammation during the saccular stage recruits macrophages and alters lung development
Source: JCI Insight. 2026 Jun 2;11(14):e193625. doi: 10.1172/jci.insight.193625 (PMC13461160; doi:10.1172/jci.insight.193625)
Supplement: Supplementary file 1 [file jciinsight-11-193625-s162.pdf]

Cell Population	Proportion of all *Tbx4* -Expressing Cells in the P5 Mouse Lung
**Lung Compartment**	
Mesenchyme	99.5%
Epithelium	0.41%
Endothelium	0.13%
**Mesenchymal Subpopulation**	
Myofibroblasts	61.03%
Wnt2 + Fibroblasts	27.92%
Adventitial Fibroblasts	0.25%
Pericytes	10.76%
Mesothlium	0.03%
Smooth Muscle Cells	0.03%
Cardiomyocyte	0.00%

$$= \frac{\left(\textbf{Number of } \textbf{\textit{Tbx4}}\textbf{-expressing cells in population}\right)}{\left(\textbf{Total number } \textbf{\textit{Tbx4}}\textbf{-expressing cells}\right)}$$

Supplemental Table 1. *Tbx4*-expressing cells are predominantly found in the mesenchyme in the P5 mouse lung. Analysis of previously published scRNA-seq of WT P5 mouse lung parenchymal cells (Negretti *et al*. 2021 [53]). Percentage of reported cell types among total *Tbx4*-expressing cells in the P5 mouse lung. Percentage calculated using the depicted equation. Definition and classification of lung cell populations as determined by original study.

Fibroblast Conditioned Media

Analyte	CTRL	IKKβ^{Tbx4}
	pg/mL media (Std dev)	pg/mL media (Std dev)
IL-1α	7.41 (7.65)	6.6 (2.69)
IL-1β	<3.2 (0)	<3.2 (0)
IL-2	<3.2 (0)	<3.2 (0)
IL-3	<3.2 (0)	<3.2 (0)
IL-4	<3.2 (0)	<3.2 (0)
IL-5	<3.2 (0)	<3.2 (0)
IL-6	3.44 (0.49)	6.89 (3.00)
LIF	<3.2 (0)	5.78 (3.17)
IL-7	<3.2 (0)	<3.2 (0)
IL-9	45.99 (13.16)	39.10 (24.03)
IL-10	<3.2 (0)	3.66 (0.62)
IL-12(p40)	3.52 (0.64)	4.28 (1.32)
IL-12(p70)	<3.2 (0)	<3.2 (0)
IL-13	<3.2 (0)	3.86 (1.32)
IL-15	3.54 (0.27)	3.75 (0.61)
IL-17	<3.2 (0)	<3.2 (0)
M-CSF (CSF1)	3.52 (0.39)	<3.2 (0)
**GM-CSF (CSF2)**	**4.49 (0.86)**	**19.72 (11.16)***
G-CSF (CSF3)	<3.2 (0)	<3.2 (0)
IFNγ	<3.2 (0)	<3.2 (0)
TNFα	<3.2 (0)	<3.2 (0)
VEGF-A	4.80 (1.97)	3.74 (1.09)
Eotaxin	<3.2 (0)	3.50 (0.60)
**CXCL1**	**<3.2 (0)**	**11.65 (3.36)***
**CXCL2**	**5.10 (3.38)**	**12.088 (2.46)***
CXCL5	5.02 (1.34)	5.78 (3.17)
CXCL9	<3.2 (0)	<3.2 (0)
**CXCL10**	**3.31 (0.22)**	**31.93 (11.81)***
**CCL2**	**9.37 (4.45)**	**91.41 (33.67)***
CCL3	3.57 (0.67)	4.44 (0.54)
CCL4	11.47 (3.65)	15.01 (2.17)
CCL5	3.26 (0.12)	6.06 (2.62)
**CCL7[A]**	**22.94 (24.13)**	**130.48 (40.76)***

Supplemental Table 2. IKKβ^{Tbx4} lung fibroblasts produce cytokines and chemokines following in vitro transgene activation. Primary lung fibroblasts were isolated from IKKβ^{Tbx4} and littermate control (CTRL) lungs on P2 and were treated with doxycycline (Dox, 3 μg/mL in media) at either passage 4 or 5. Conditioned media was gathered from fibroblast cultures after 24 h and analyzed for cytokine and chemokine expression. Data are expressed as mean with SD. n = 3 biological replicates per group; [A] analyte measured using ELISA; bold analytes demonstrate statistically significant differences between groups, * $P < 0.05$ by unpaired 2-tailed *t* test.

P5 Lung Lysate

Analyte	CTRL	IKKβ^{Tbx4}
	pg/mg total protein (Std dev)	pg/mg total protein (Std dev)
IL-1α	186.34 (318.29)	40.78 (32.26)
**IL-1β**	**<3.2 (0)**	**7.77 (3.93)***
IL-2	0.91 (.39)	0.75 (0.35)
IL-3	<3.2 (0)	<3.2 (0)
IL-4	<3.2 (0)	<3.2(0)
IL-5	104.52 (45.0)	157.77 (61.28)
**IL-6**	**<3.2 (0)**	**43.07 (27.94)***
**LIF**	**<3.2 (0)**	**25.39 (15.23)***
IL-7	<3.2 (0)	<3.2 (0)
IL-9	63.70 (22.62)	48.04 (22.13)
IL-10	5.06 (2.01)	7.11 (3.14)
IL-12(p40)	<3.2 (0)	4.20 (1.32)
IL-12(p70)	18.19 (7.07)	15.20 (6.73)
IL-13	22.90 (2.76)	43.65 (25.52)
IL-15	3.98 (1.06)	6.82 (3.08)
IL-17	<3.2 (0)	3.61 (2.53)
**M-CSF (CSF1)**	**3.51 (0.65)**	**70.53 (51.24)***
**GM-CSF (CSF2)**	**2.82(0.63)**	**20.82 (13.10)***
G-CSF (CSF3)	4.16 (2.05)	868.87 (970.97)
IFNγ	19.16 (13.21)	36.95 (34.64)
TNFα	<3.2 (0)	2.53 (1.14)
**VEGF-A**	**236.67 (43.43)**	**148.81 (51.29)***
Eotaxin	331.71 (56.85)	374.16 (189.86)
**CXCL1**	**30.02 (7.57)**	**793.15 (356.53)***
CXCL2	14.89 (2.60)	138.45 (120.69)
CXCL5	23.30 (9.82)	326.77 (433.172)
**CXCL9**	**17.49 (3.85)**	**153.43 (102.15)***
**CXCL10**	**32.05 (7.41)**	**565.04 (312.20)***
**CCL2**	**15.15 (3.53)**	**2063.94 (1216.37)***
CCL3	25.56 (8.69)	45.34 (24.97)
CCL4	35.27 (11.92)	42.55 (19.83)
**CCL5**	**3.14 (1.05)**	**975.45 (653.64)***
**CCL7^{A}**	**9.96 (3.05)**	**1499.49 (1014.97) ***

Supplemental Table 3. Cytokines and chemokines are increased in IKKβ^{Tbx4} lungs on P5. Doxycycline was added to drinking water of lactating dams from P0-P5. Lungs from control (CTRL) and IKKβ^{Tbx4} mice were harvested on P5 and cytokines and chemokines were quantified. Inflammatory protein concentrations were normalized to total protein content. Data are expressed as mean with SD. n = 5 mice per group; ^{A} analyte measured using ELISA; bold analytes demonstrate statistically significant differences between groups, * $P < 0.05$ by unpaired 2-tailed *t* test.

Target Antigen	Clone	Fluorochrome	Manufacturer
**Flow Cytometry**			
CAR4	polyclonal (# AF2414)	N/A, Unconjugated	R&D Systems
CCR2	SA203G11	PE-Cy5	Biolegend
CD11b	M1/70	BV650	Biolegend
CD11c	N418	PE	Biolegend
CD31 (PECAM)	390	PerCP-Cy5	Biolegend
CD45	30-F11	BV510	Biolegend
CD45	30-F11	FITC	Biolegend
CD117 (KIT)	2B8	APC-Cy7	Biolegend
CD140a (PDGFR-α)	APA5	APC	Biolegend
CD140b (PDGFR-β)	APB5	PE-Cy7	Invitrogen
CD144 (VE-Cadherin)	11D4.1	BV786	BD Biosciences
CD146	ME-9F1	BV510	BD Biosciences
EpCAM	390	FITC	Biolegend
F4/80	BM8	FITC	Biolegend
MERTK	2B10C42	APC	Biolegend
TER-119	TER-119	FITC	Biolegend
**FACS**			
CD31 (PECAM)	390.00	PE-Cy7	Biolegend
CD45	30-F11	BV510	Biolegend
EpCAM	G8.8	APC-Cy7	Biolegend
TER-119	TER-119	APC	Biolegend

Supplemental Table 4. Primary antibodies for flow cytometry and FACS.

Gene	Forward (Sense)	Reverse (Anti-sense)
Tnf	*5′-GACCCTCACACTCAGATCATCTTCT-3′*	*5′-CCACTTGGTTTGCTACGA-3′*
Il1b	*5′-ACAGAATATCAACCAACAAGTGATATTCTC-3′*	*5′-GATTCTTTCCTTTGAGGCCCA-3′*
Il6	*5′-AAGAGACTTCCATCCAGTTGCC-3′*	*5′-TGAAGTCTCCTCTCCGGACT-3′*
Il10	*5′-ACCTGCTCCACTGCCTTGCT-3′*	*5′-GGTTGCCAAGCCTTATCGGA-3′*
Il12a	*5′-TGGACCTGCCAGGTGTCTTAG-3′*	*5′-CAATGTGCTGGTTTGGTCCC-3′*
Cxcl1	*5′-CCGAAGTCATAGCCACACTCAA-3′*	*5′-GCAGTCTGTCTTCTTTCTCCGTTAC-3′*
Cxcl10	*5′-CCGAAGTCATAGCCACACTCAA-3′*	*5′-GCAGTCTGTCTTCTTTCTCCGTTAC-3′*
Ccl2	*5′-TGGCTCAGCCAGATGCAG-3′*	*5′-GGTGATCCTCTTGTAGCTCTCCAG-3′*
Ccl3	*5′-TGCCCTTGCTGTTCTTCTCT-3′*	*5′-GATGAATTGGCGTGGAATCT-3′*
Ccl7	*5′-GCTTTCAGCATCCAAGTGTG-3′*	*5′-GGTGATCCTTCTGTAGCTCTTG-3′*
Ccl8	*5′-CATGGAGATCCTTGACCAGAAG-3′*	*5′-CTCTCTGCCTGGAGAAGATTAG-3′*
Ccl12	*5′-CTTCTATGCCTCCTGCTCATAG-3′*	*5′-GGACGTGAATCTTCTGCTTA-3′*
Csf2	*5′-GAAGATATTCGAGCAGGGTCTAC-3′*	*5′-CTTGTGTTTCACAGTCCGTTTC-3′*
Csf3	*5′-AACTTTGCCACCACCATCT -3′*	*5′-GCTGGAAGGCAGAAGTGAA -3′*
Acta2	*5′-GACTCTCTTCCAGCCATCTTTC-3′*	*5′-GAC AGG\ACGTTGTTAGCATAGA-3′*
Tgfbi	*5′-CTCCCTTCAGGACATCCATAAC-3′*	*5′-GCATTGCTGCCCATGATAAG-3′*
Fbln5	*5′-TGTCAACACCTATGGCTCTTTC-3′*	*5′-GAAGCTGCACTCGTCCATATC-3′*
Eln	*5′-CTGCCAAAGCTGCCAAATAC-3′*	*5′-CCAACACCATAGCCAGGAAA-3′*
Loxl1	*5′-CTATGACCTCCGAGTGCTATTG-3′*	*5′-TCGTCCATGCTGTGGTAATG-3′*
Mmp9	*5′-TGCACTGGGCTTAGATCATTC -3′*	*5′-TGCCGTCTATGTCGTCTTTATTC-3′*
Vegfa	*5′- AGGCTGCTGTAACGATGAAG- 3′*	*5′-TCTCCTATGTGCTGGCTTTG-3′*
Kdr	*5′-GCGGAGACGCTCTTCATAATA-3′*	*5′-GACAAGAAGGAGCCAGAAGAA-3′*
Angpt1	*5′-CACAGTACGACAGATTCCACATAG-3′*	*5′-GAAATCGGCACCGTGTAAGA-3′*
Tek	*5′-CCGGCATGAAGTACCTGATATT -3′*	*5′-GGTGAACAGGTTTCCTCCTATG -3′*
Actb	*5′-CTCCCTGGAGAAGAGCTATGA-3′*	*5′-CCAAGAAGGAAGGCTGGAAA-3′*
Ikbkb (Human)	*5′-GAC GCC ATC CAC GCT GTT TTG-3′*	*5′-CTT CTC ATG ATC TGG ATC TCC-3′*

Supplemental Table 5. Primer sequences. Primers were designed using PrimerQuest design tool (Integrated DNA Technologies, Coralville, IA).

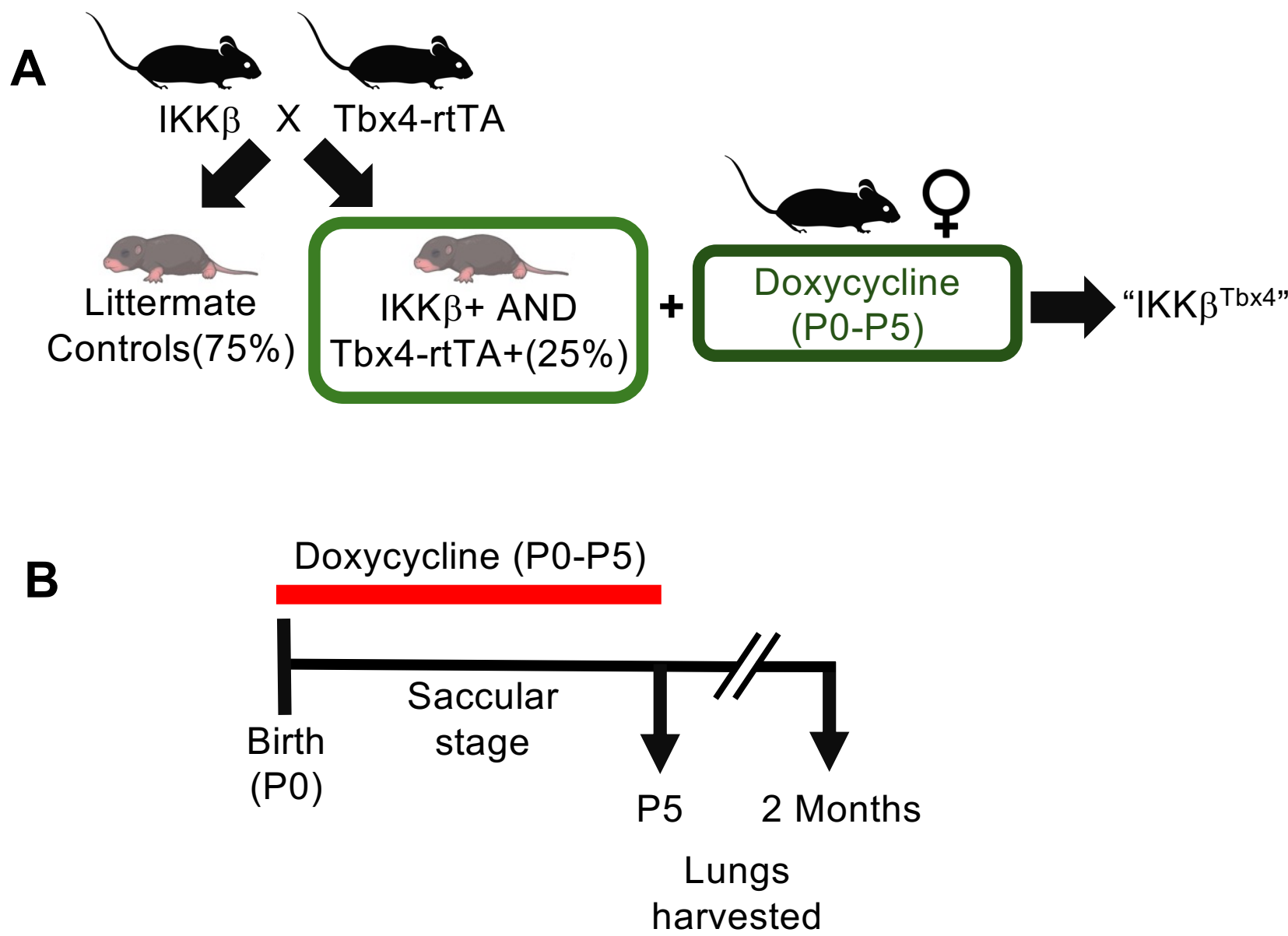

Supplemental Figure 1. "IKKβ^{Tbx4}" mice are a model of saccular stage lung mesenchymal inflammation. **A:** Schematic of the mating strategy used to breed transgenic IKKβ^{Tbx4} and littermate control mice. Hemizygous IKKβ mice were crossed with heterozygous Tbx4-rtTA mice. Lactating dams were given doxycycline to induce expression of the IKKβ transgene in *Tbx4* lung enhancer-positive cells in pups carrying both transgenes. Mice positive for both transgenes with active IKKβ expression are referred to as "IKKβ^{Tbx4}" mice. **B:** Schematic of experimental timepoints. To induce transgene expression in double transgenic mice during the saccular stage, Dox was added to drinking water of dams from P0-P5. Lungs were then harvested from pups on P5 or at 2 mo of age.

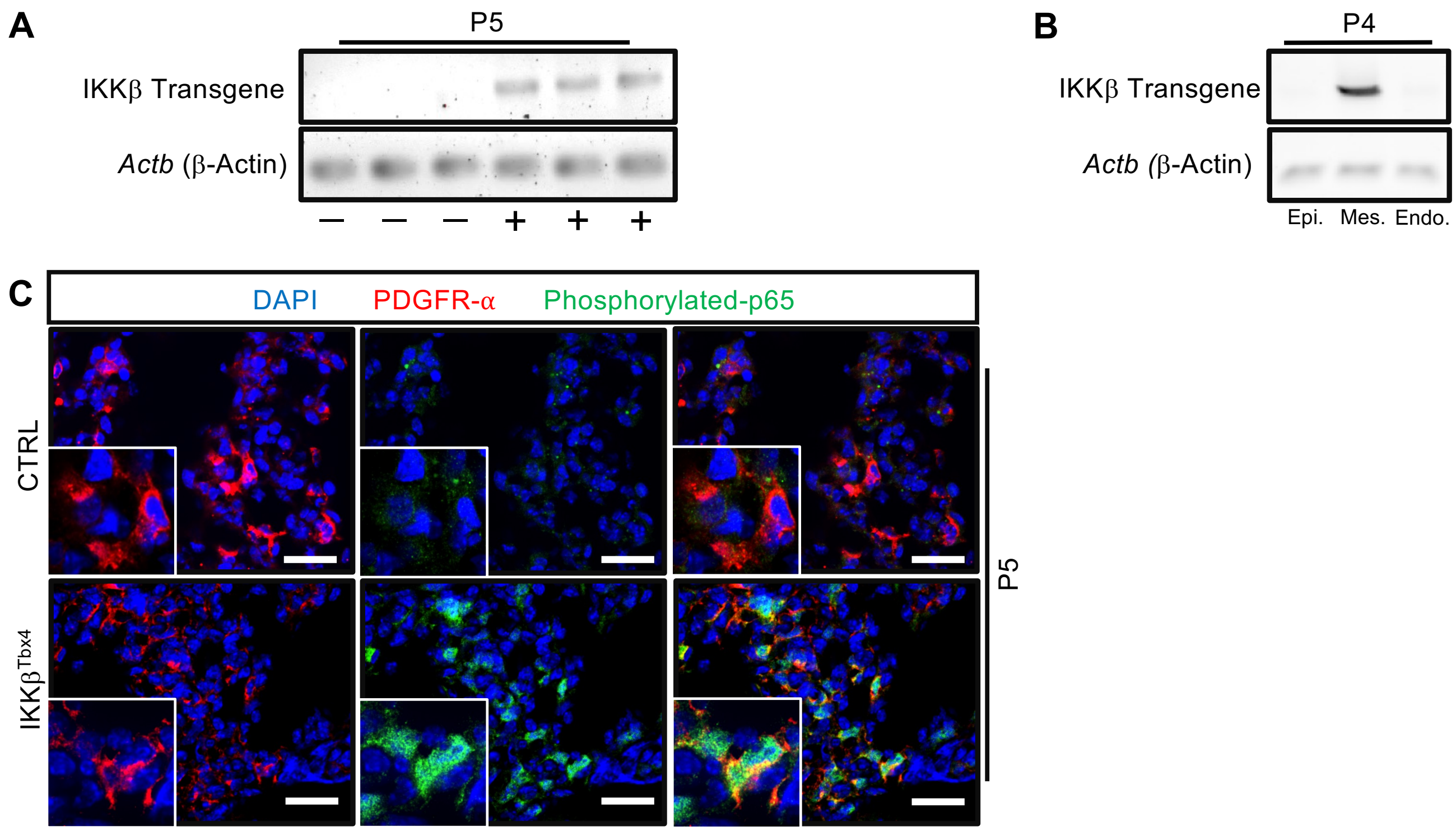

Supplemental Figure 2. NF-κB activation in the lung mesenchyme of IKKβ^{Tbx4} mice. **A-C:** Doxycycline (Dox) was added to drinking water of lactating dams beginning P0 and continued until lung harvest during the saccular stage. **A:** Lungs of Dox-exposed IKKβ^{Tbx4} (+) and littermate control (-) mice were harvested at P5 to quantify mRNA expression of the activated human IKKβ transgene and *Actb* using PCR. Product was visualized using agarose gel electrophoresis. **B:** Lungs of Dox-exposed IKKβ^{Tbx4} mice were harvested at P4 and processed for FACS. Lung parenchymal cells were sorted into epithelial (“Epi.” [CD45^{neg}, EpCAM^{pos}, PECAM^{neg}]), mesenchymal (“Mes.” [CD45^{neg}, EpCAM^{neg}, and PECAM^{neg}]), and endothelial (“Endo.” [CD45^{neg}, EpCAM^{neg}, PECAM^{pos}]) cell populations. mRNA expression of the activated human IKKβ transgene and *Actb* was quantified in transgenic lung parenchymal isolates using PCR. Product was visualized using agarose gel electrophoresis. **C:** Representative photomicrographs of lung sections from control (CTRL) and IKKβ^{Tbx4} mice at P5 immunostained for fibroblast marker PDGFR-α and phosphorylated-p65, a marker of NF-κB activity. Fluorescent imaging was performed using confocal microscopy. Scale bar = 100 μm.

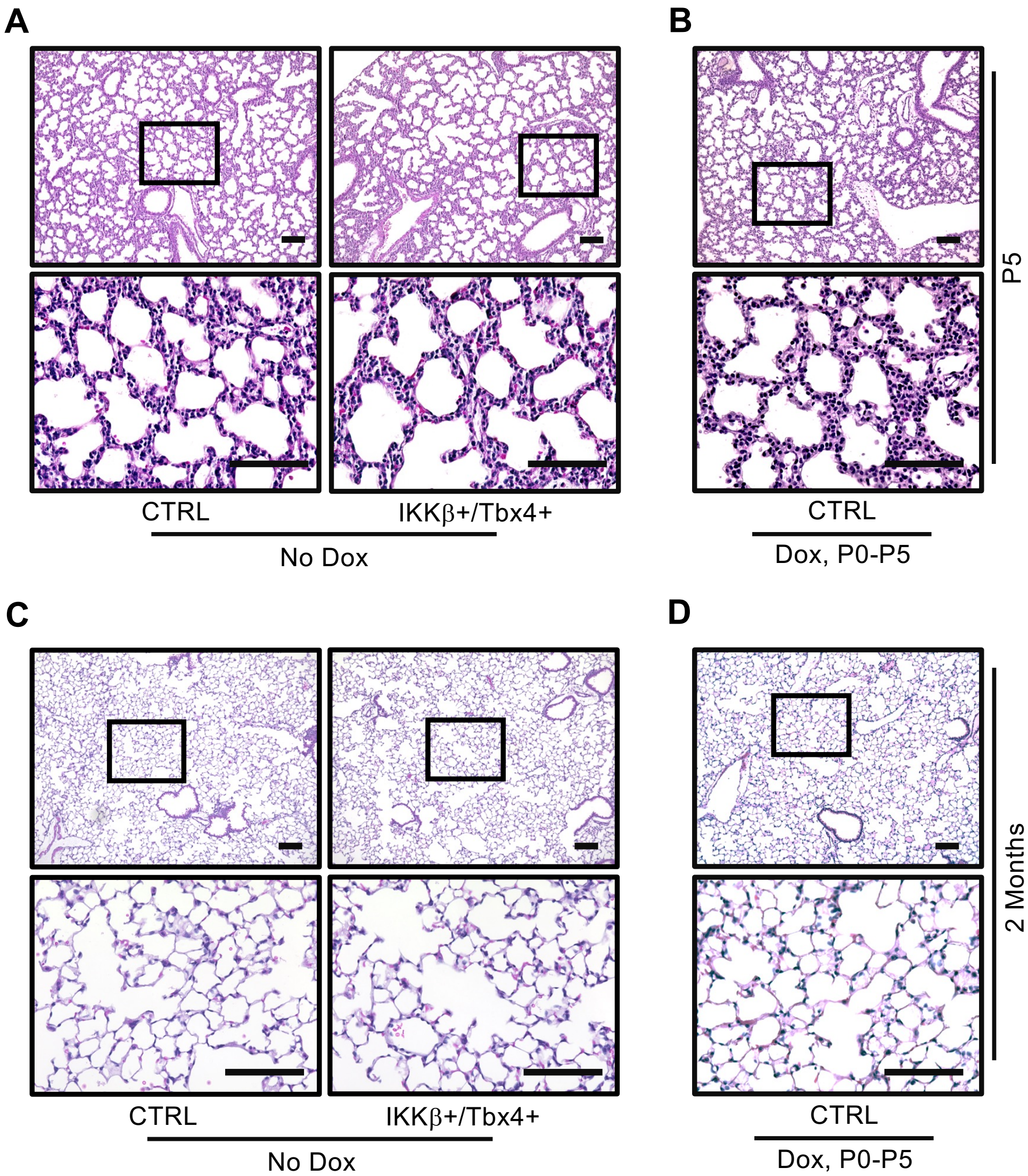

Supplemental Figure 3. Effect of doxycycline (Dox) on the saccular stage lung. **A&C:** Littermate control (CTRL) mice and mice carrying both IKKβ and Tbx4-rtTA transgenes ("IKKβ+/Tbx4+") with no Dox exposure were harvested at P5 **(A)** and 2 mo of age **(C). B&D:** CTRL mice were treated with Dox from P0-P5 and lungs were harvested at P5 **(B)** and 2 mo of age **(D)**. Representative H&E-stained lung sections, **A-D**; scale bar = 100 μm **(A-D)**.

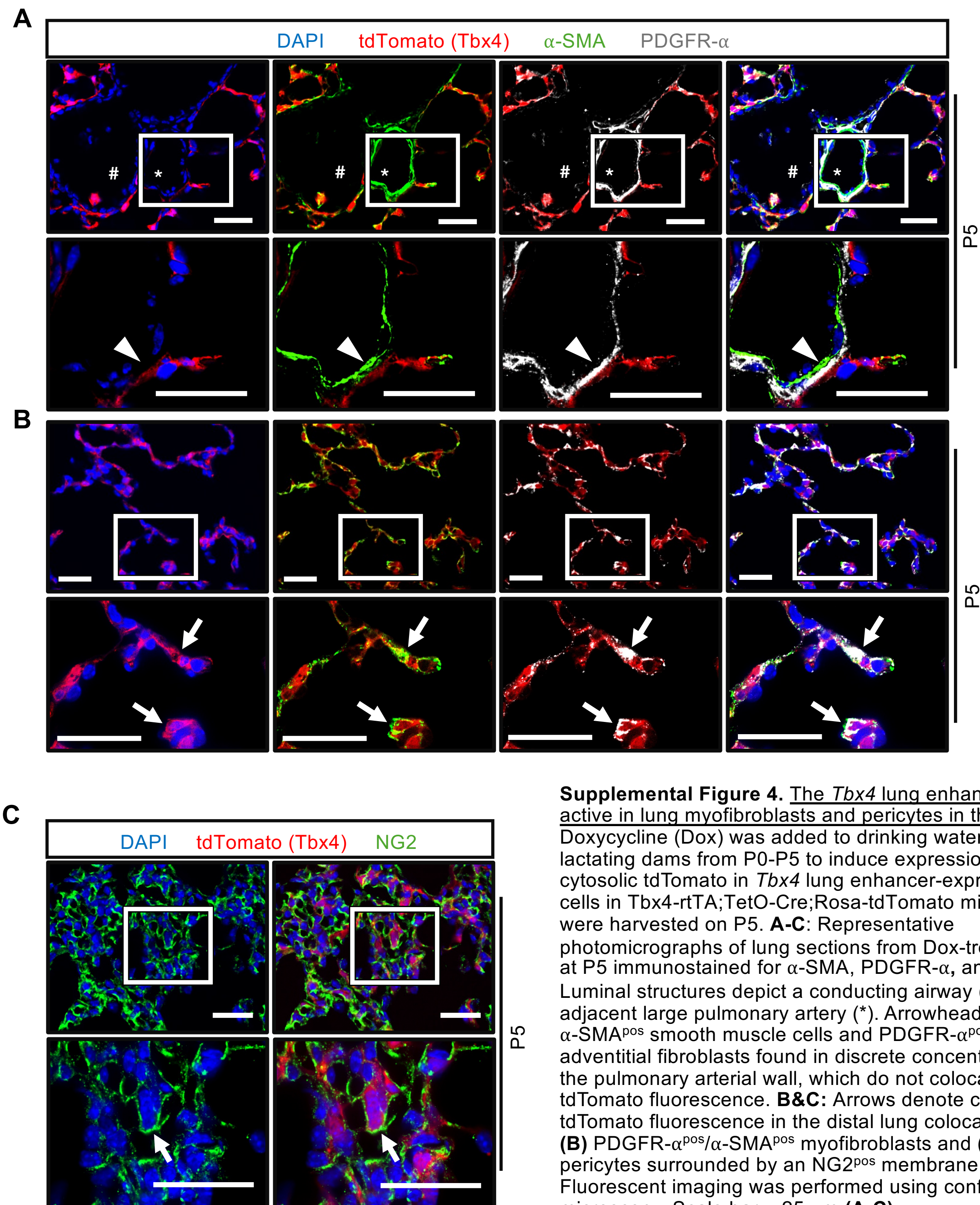

Supplemental Figure 4. The *Tbx4* lung enhancer is active in lung myofibroblasts and pericytes in the P5 lung. Doxycycline (Dox) was added to drinking water of lactating dams from P0-P5 to induce expression of cytosolic tdTomato in *Tbx4* lung enhancer-expressing cells in Tbx4-rtTA;TetO-Cre;Rosa-tdTomato mice. Lungs were harvested on P5. **A-C**: Representative photomicrographs of lung sections from Dox-treated mice at P5 immunostained for α-SMA, PDGFR-α**,** and NG2. **A:** Luminal structures depict a conducting airway (#) and an adjacent large pulmonary artery (*). Arrowheads highlight α-SMA^{pos} smooth muscle cells and PDGFR-α^{pos} adventitial fibroblasts found in discrete concentric layers in the pulmonary arterial wall, which do not colocalize with tdTomato fluorescence. **B&C:** Arrows denote cytosolic tdTomato fluorescence in the distal lung colocalizing with **(B)** PDGFR-α^{pos}/α-SMA^{pos} myofibroblasts and **(C)** pericytes surrounded by an NG2^{pos} membrane. Fluorescent imaging was performed using confocal microscopy. Scale bar = 25 µm **(A-C)**.

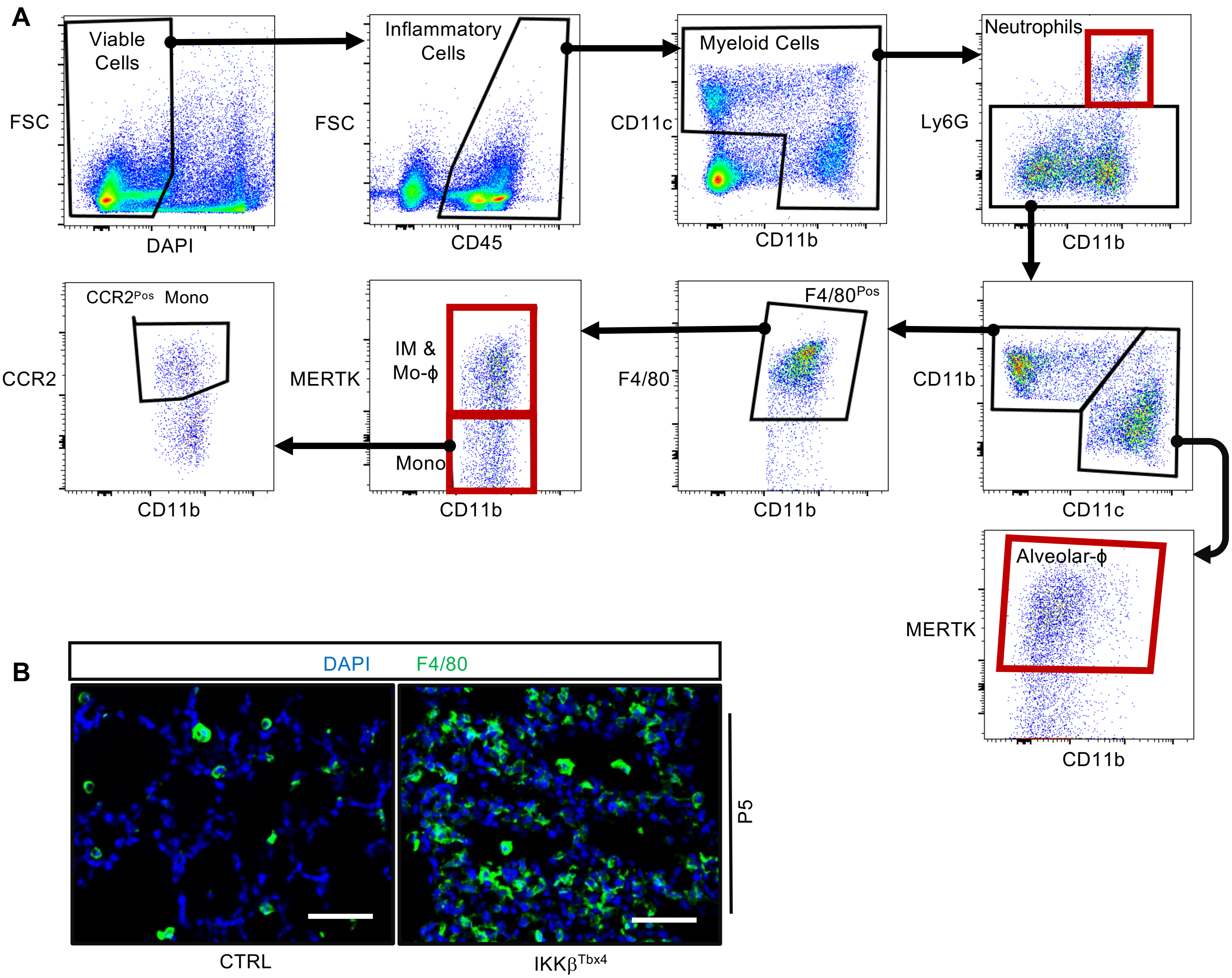

Supplemental Figure 5. Characterization of myeloid populations in control (CTRL) and $IKK\beta^{Tbx4}$ mouse lungs on P5. **A:** Contour plots depicting gating strategy used to identify distinct myeloid cell populations in the P5 lung. Red boxes reflect myeloid population represented in **Figure 2B** (Forward Scatter=“FSC”, Macrophage=“ϕ”, interstitial and monocyte-derived macrophages = “IM & Mo-ϕ”, monocytes = “Mono”). **B:** Doxycycline was added to drinking water of lactating dams from P0-P5 and lungs were harvested on P5. Representative photomicrographs of P5 littermate control (CTRL) and $IKK\beta^{Tbx4}$ lung sections immunostained for macrophage/monocyte marker F4/80. $IKK\beta^{Tbx4}$ lungs demonstrated marked F4/80 localization to the lung interstitium, relative to CTRL. Fluorescent imaging was performed using confocal microscopy. Scale bar = 100 µm.

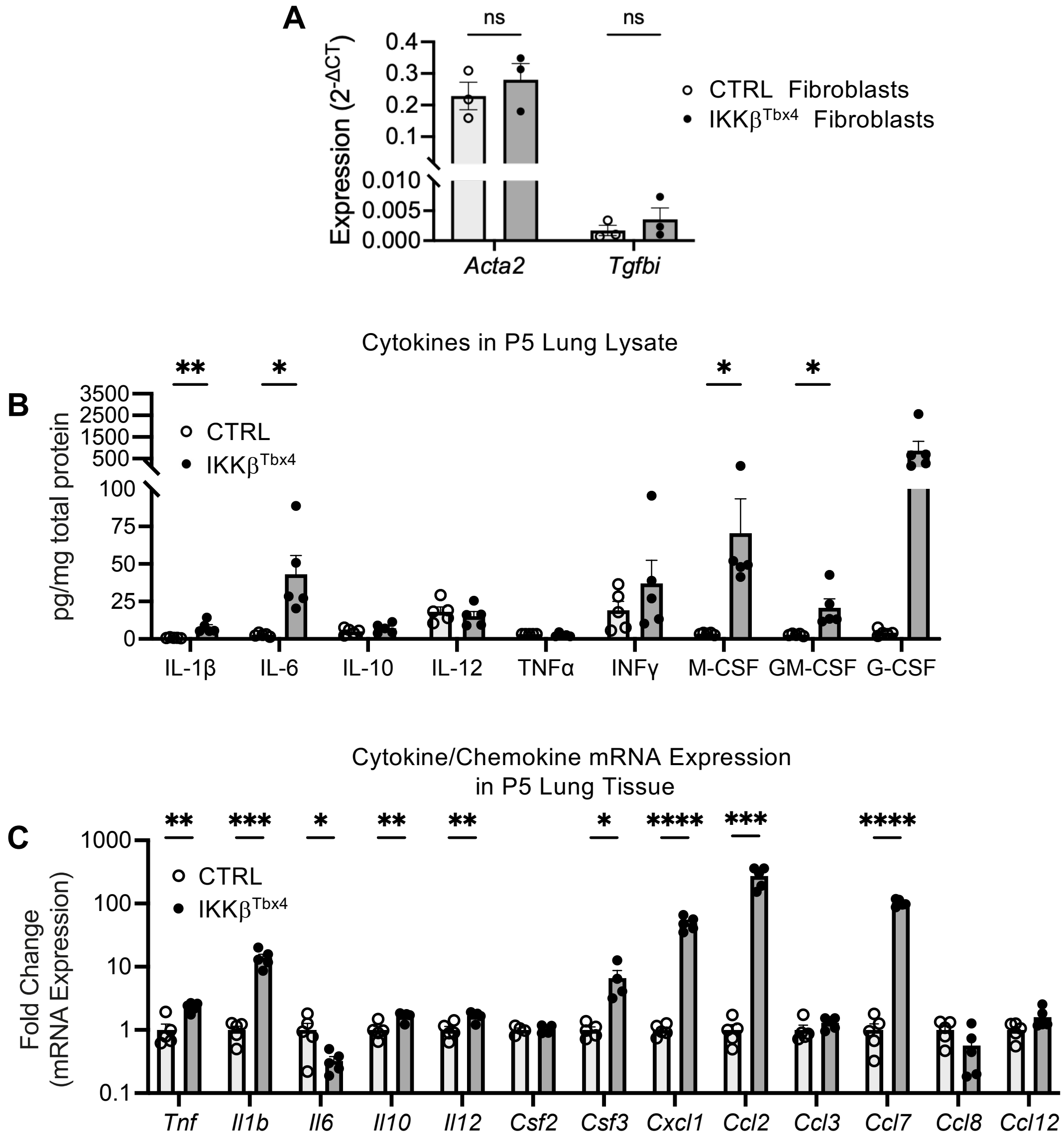

Supplemental Figure 6. Mesenchymal IKKβ expression increases the production of inflammatory cytokines and chemokines. **A:** Primary lung fibroblasts were isolated from littermate control (CTRL) and IKKβ^{Tbx4} mice on P2 and passaged 4 or 5 times. Cells were treated with doxycycline (Dox, 3 μg/mL in media) for 24 h before cell harvest and RNA isolation. qRT-PCR was done to examine myofibroblast markers; ns = not significant. **B&C:** Dox was added to drinking water of lactating dams from P0-P5 and lungs from CTRL and IKKβ^{Tbx4} mice were harvested at P5. **B:** Cytokine production was quantified in P5 lung lysate using a 32-plex cytokine/chemokine multiplex assay. Lysate protein concentrations were normalized to total protein content of the sample. Full multiplex results are shown in "**Supplemental Table 3**." **C:** Relative gene expression of select cytokines and chemokines in P5 IKKβ^{Tbx4} lungs compared to CTRL measured by qRT-PCR. Data are expressed as mean ± SEM; n=3 mice per group **(A)**; n= 5 mice per group **(B&C)**; * $P < 0.05$, ** $P < 0.01$, *** $P < 0.001$, and **** $P < 0.0001$ by unpaired 2-tailed *t* test **(A-C)**.

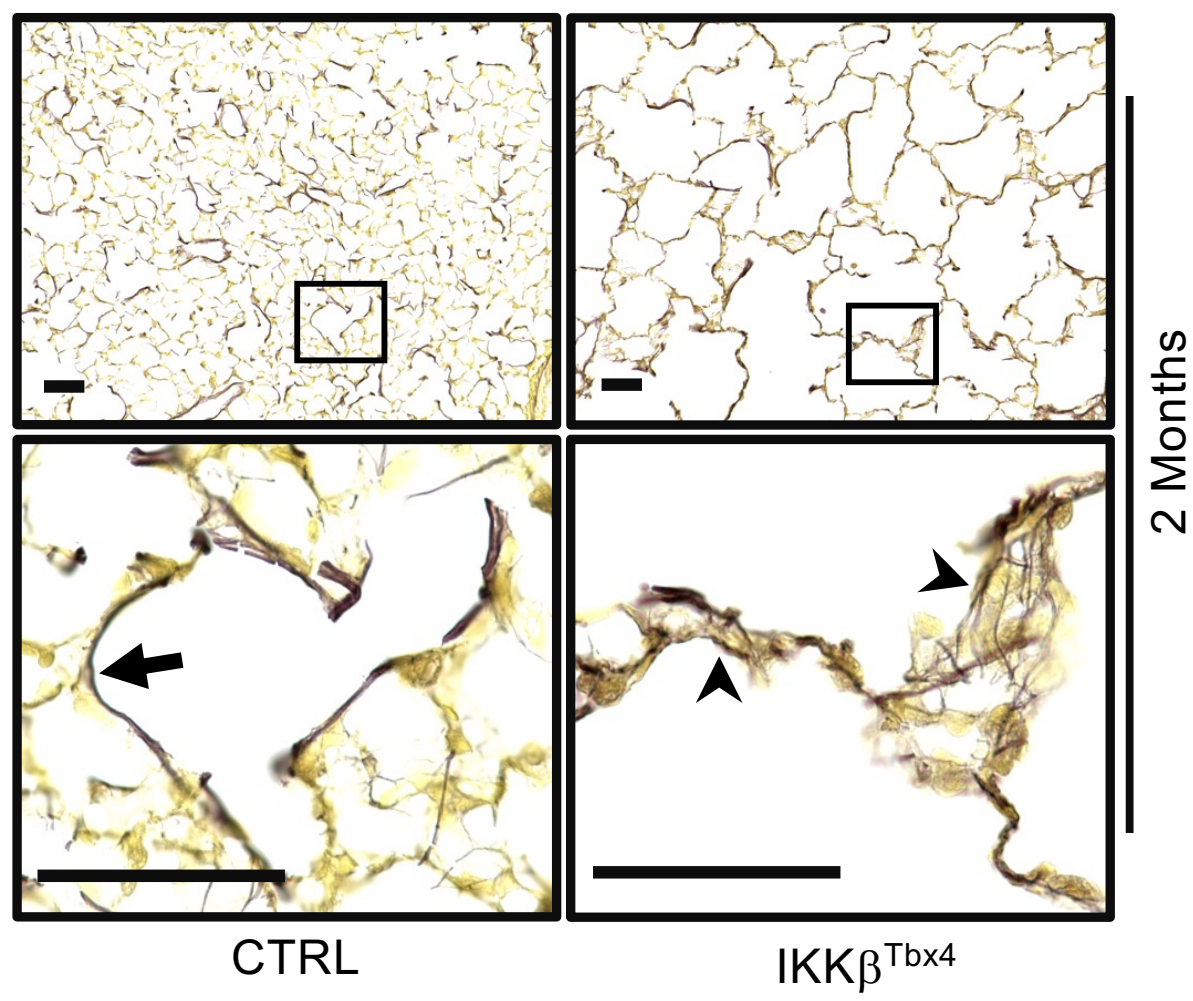

Supplemental Figure 7. Mouse lungs exposed to mesenchymal-derived inflammation during the saccular stage demonstrate long-term abnormalities in elastin organization. Doxycycline was added to drinking water of lactating dams from P0-P5 and lungs were harvested at 2 mo of age. Representative Hart's-stained sections from littermate control (CTRL) and $IKK\beta^{Tbx4}$ lungs. Arrow indicates elastic fiber deposition in the alveolar wall; arrowheads denote disorganized elastic fibers in $IKK\beta^{Tbx4}$ lungs. Experiment was repeated 4-5 times per group; Scale bar = 100 μm.

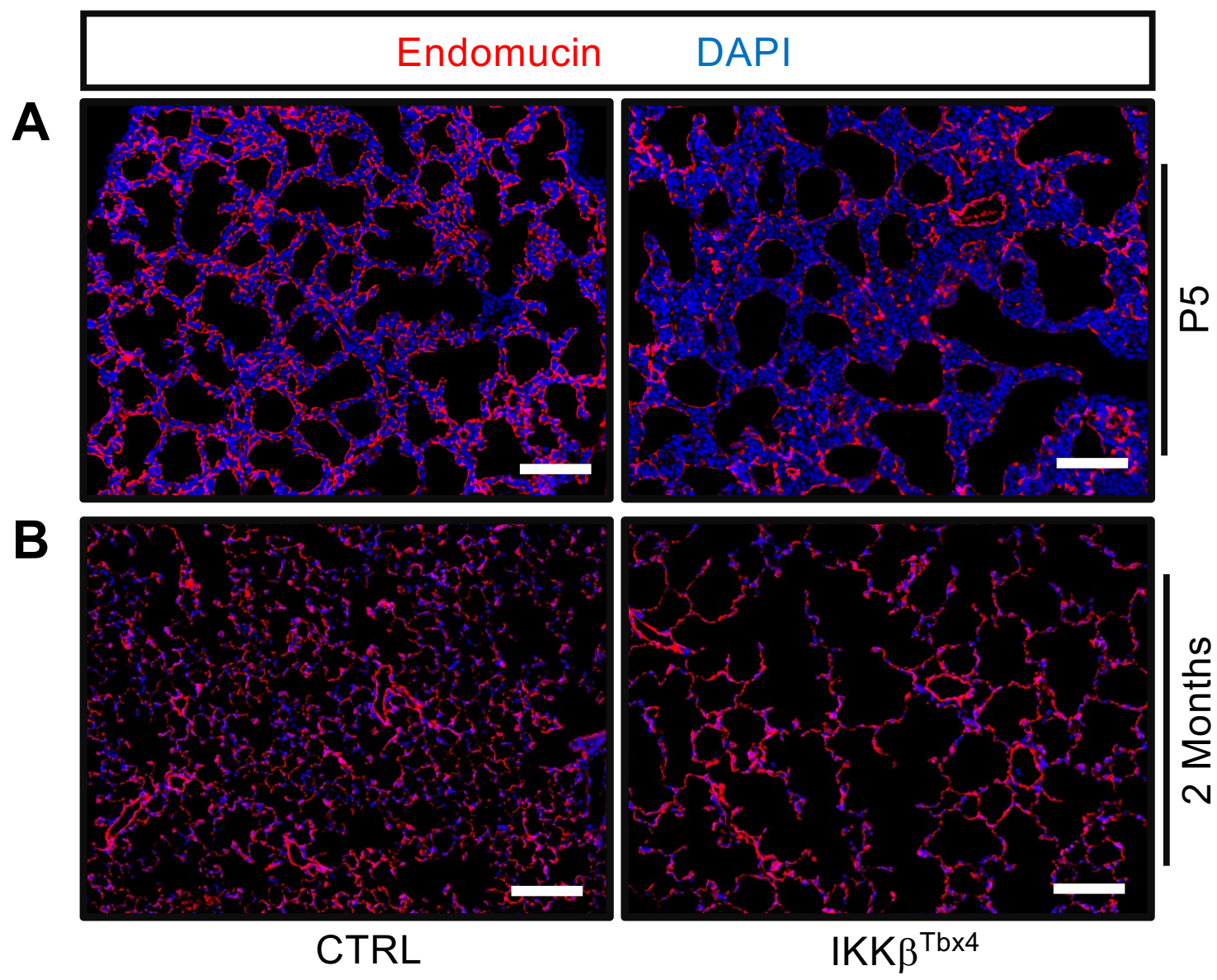

Supplemental Figure 8. IKKβ^{Tbx4} mouse lungs have abnormal microvascular structure at P5 and 2 mo of age. Doxycycline was added to drinking water of lactating dams from P0-P5 and lungs were harvested at P5 or 2 mo of age. **A&B:** Representative photomicrographs of lung sections from littermate control (CTRL) and IKKβ^{Tbx4} mice at P5 **(A)** or 2 mo of age **(B)** immunostained for Endomucin, a marker of capillaries and veins. Nuclei are labeled with DAPI. Fluorescent imaging was performed using widefield microscopy. Experiment was repeated 4-5 times per group; Scale bar = 100 μm.

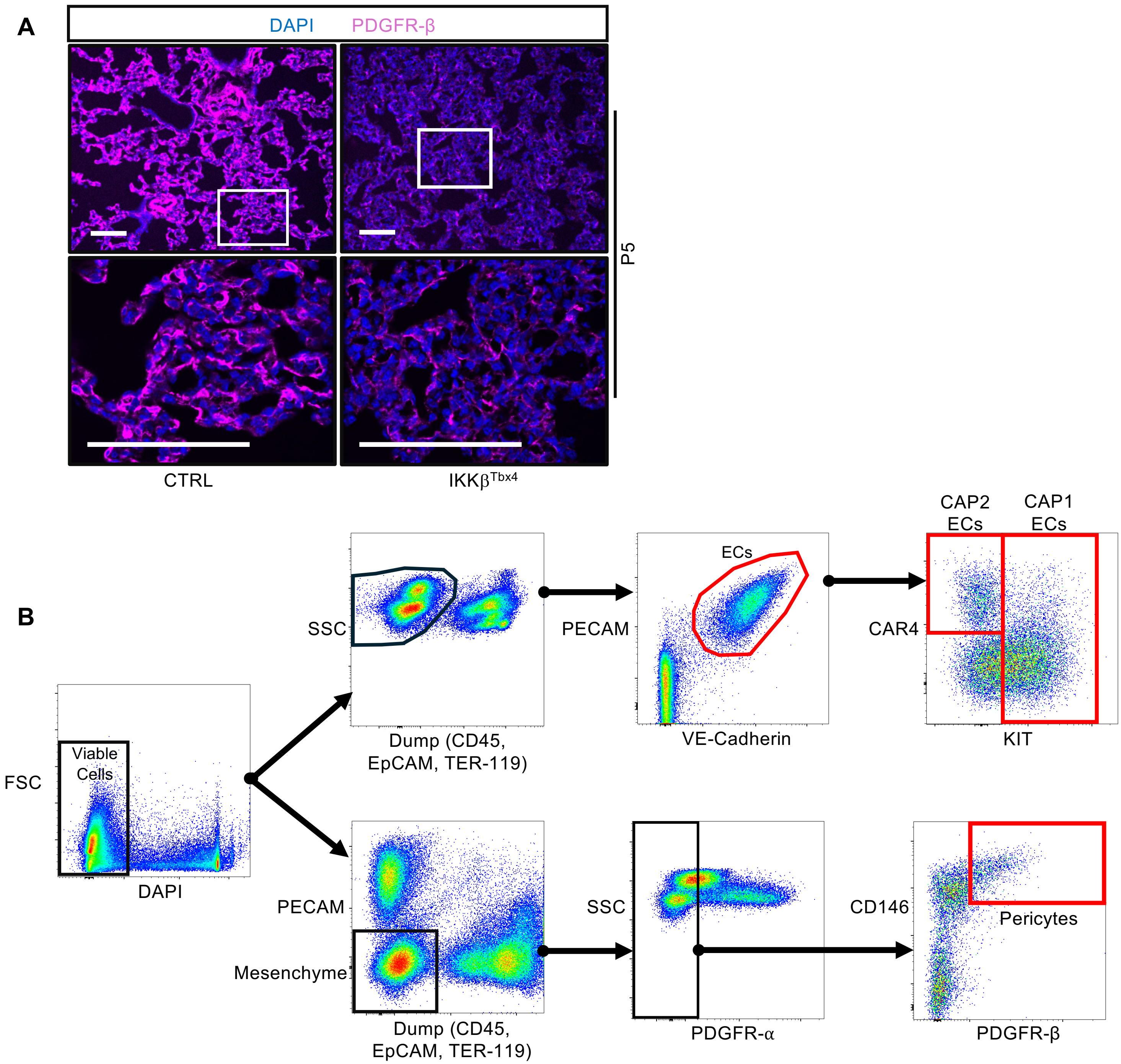

Supplemental Figure 9. Characterization of lung microvasculature populations in the P5 IKKβ^{Tbx4} mouse lung. **A:** Representative photomicrographs of lung sections from control (CTRL) and IKKβ^{Tbx4} lungs at P5 immunostained for pericyte marker PDGFR-β. Fluorescent imaging was performed using confocal microscopy. Scale bar = 100μm. **B:** Contour plots depicting gating strategy used to identify and quantify total endothelial cells (ECs), CAP1 and CAP2 ECs, and pericytes in the P5 lung (Forward ["FSC"] and Side Scatter ["SSC"]). Red boxes reflect populations represented in **Figure 6F-I**.

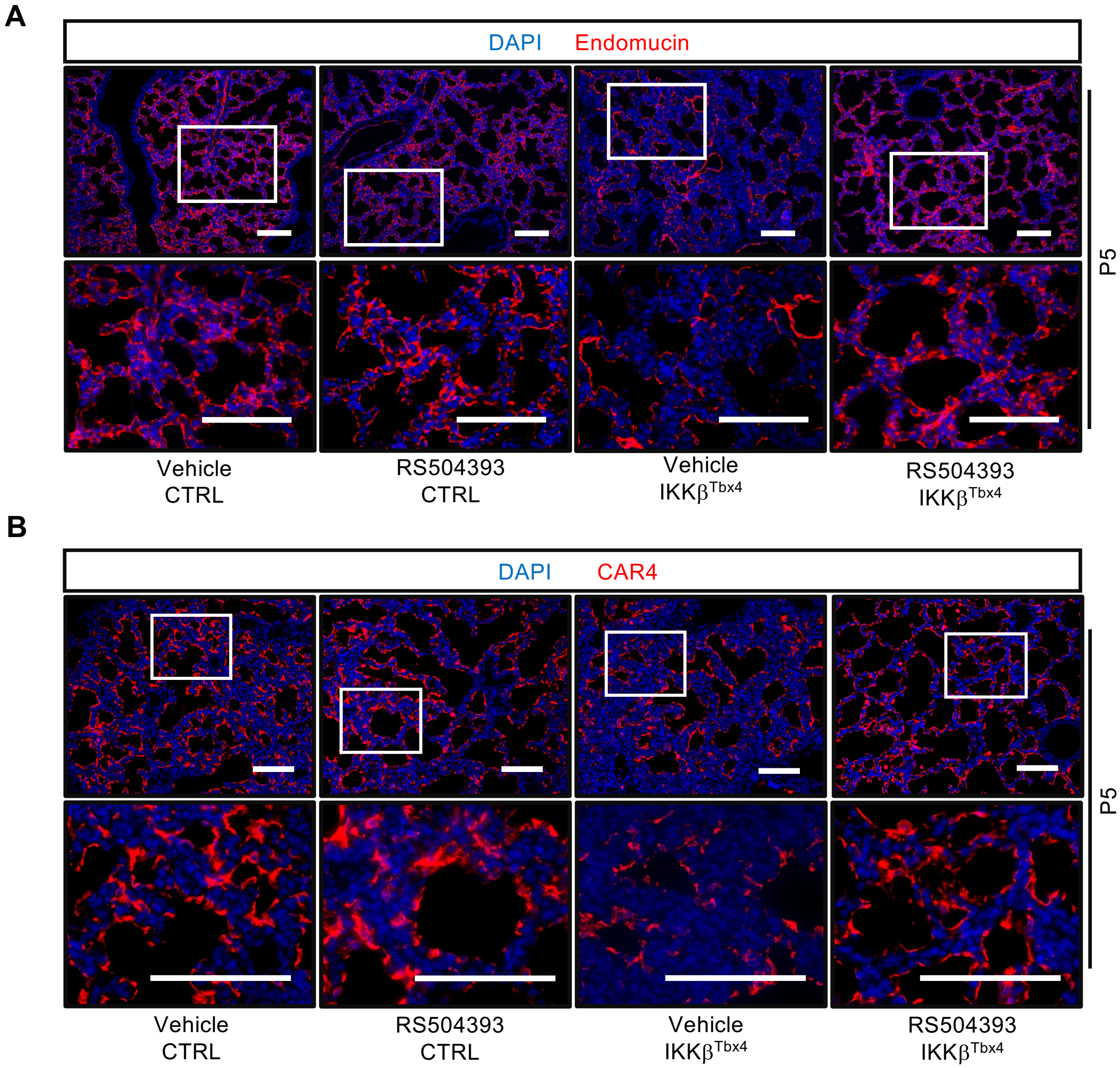

Supplemental Figure 10. CCR2 antagonism rescues microvascular development in IKKβ^{Tbx4} lungs. Doxycycline was added to drinking water of lactating dams during the saccular stage from P1-P5 and i.p. injections of either vehicle (20μl of DMSO) or CCR2 antagonist (RS504393, 2μg/g) were given daily P1-P4. Lungs were harvested on P5. **A&B:** Representative photomicrographs of lung sections from control (CTRL) and IKKβ^{Tbx4} mice at P5 immunostained for Endomucin, a marker of lung capillaries and veins, and CAP2 endothelial cell marker CAR4. Nuclei are labeled with DAPI. Fluorescent imaging was performed using widefield microscopy. Experiment was repeated 4-5 times per group; Scale bar = 100 μm (**A&B)**.
