# Supplementary material for: Mesenchyme-derived inflammation during the saccular stage recruits macrophages and alters lung development
Source: JCI Insight. 2026 Jun 2;11(14):e193625. doi: 10.1172/jci.insight.193625 (PMC13461160; doi:10.1172/jci.insight.193625)
Supplement: Supplementary file 2 [file jciinsight-11-193625-s163.pdf]

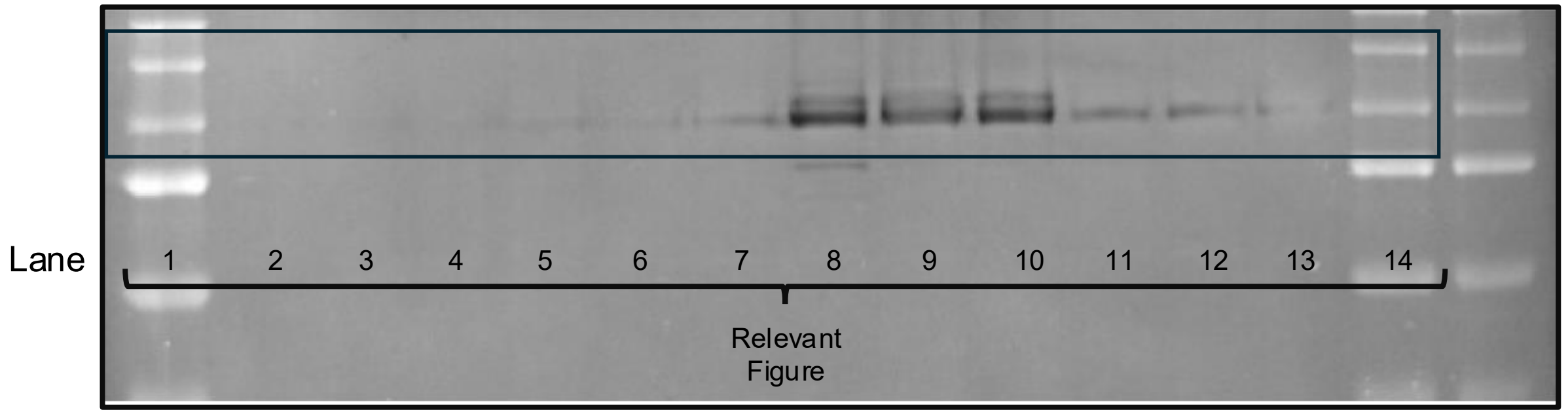

- Full Unedited Polyacrylamide-Collagen Gel for Figure 5H
    - Embedded black box depicts portion of image used in the figure

- MMP9 Zymography:
    - Lane 1&14: Ladders
    - Lane 2-4: DMSO-Treated Control Mouse Lung Lysate
    - Lane 5-7: RS504393-Treated Control Mouse Lung Lysate
    - Lane 8-10: DMSO-Treated IKKβ^{Tbx4} Mouse Lung Lysate
    - Lane 11-13: RS504393-Treated IKKβ^{Tbx4} Mouse Lung Lysate

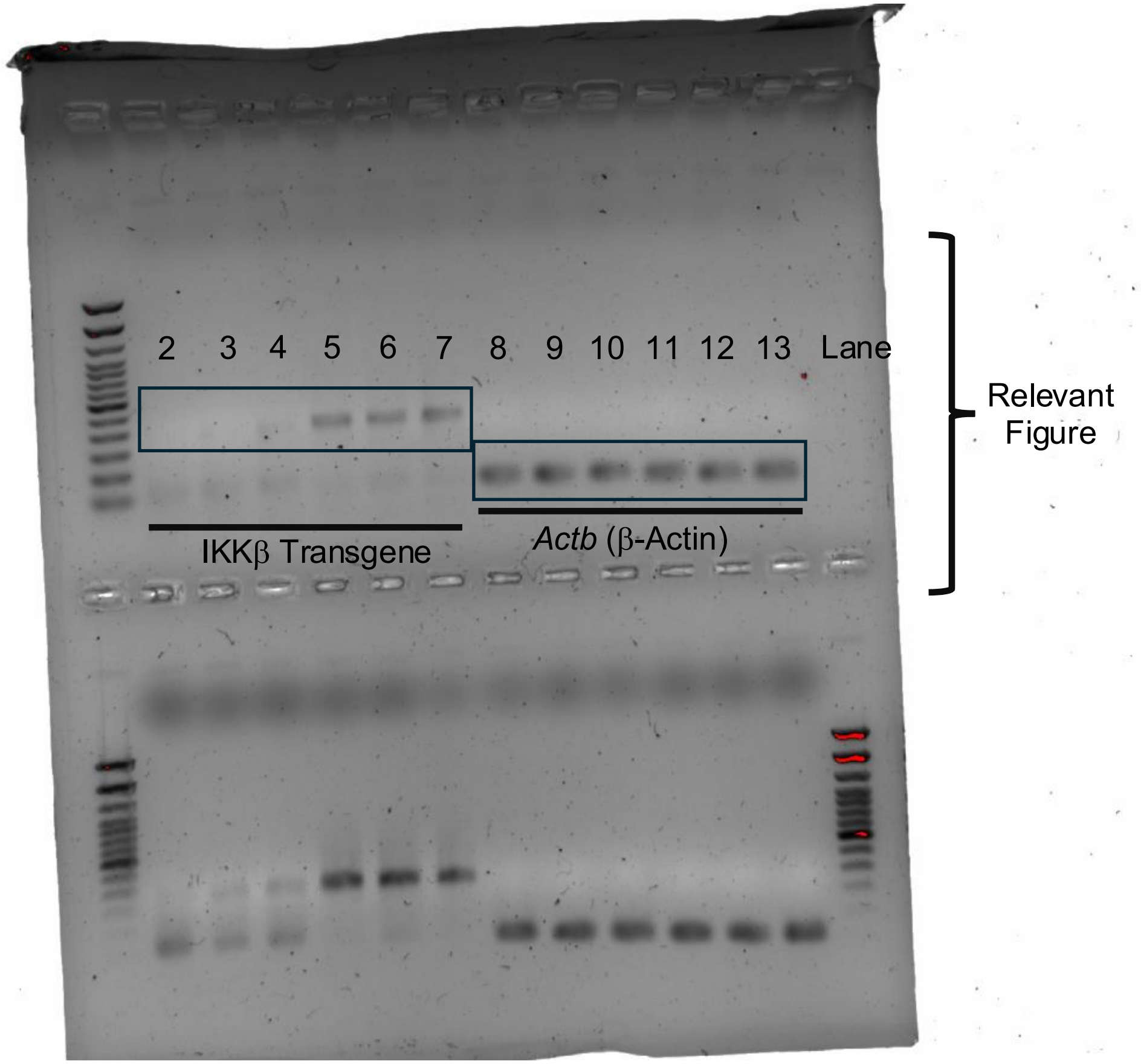

- Full Unedited Agarose Gel for Supplemental Figure 2A
  - Embedded black boxes depict portions of image used in the figure

- Amplified PCR products, IKKβ Transgene (Lanes 2-7), *Actb* (β-Actin, Lanes 8-13)
  - Control Lung, Lanes 2-4 and 8-10
  - IKKβ^{Tbx4} Lung, Lanes 5-7 and 11-13

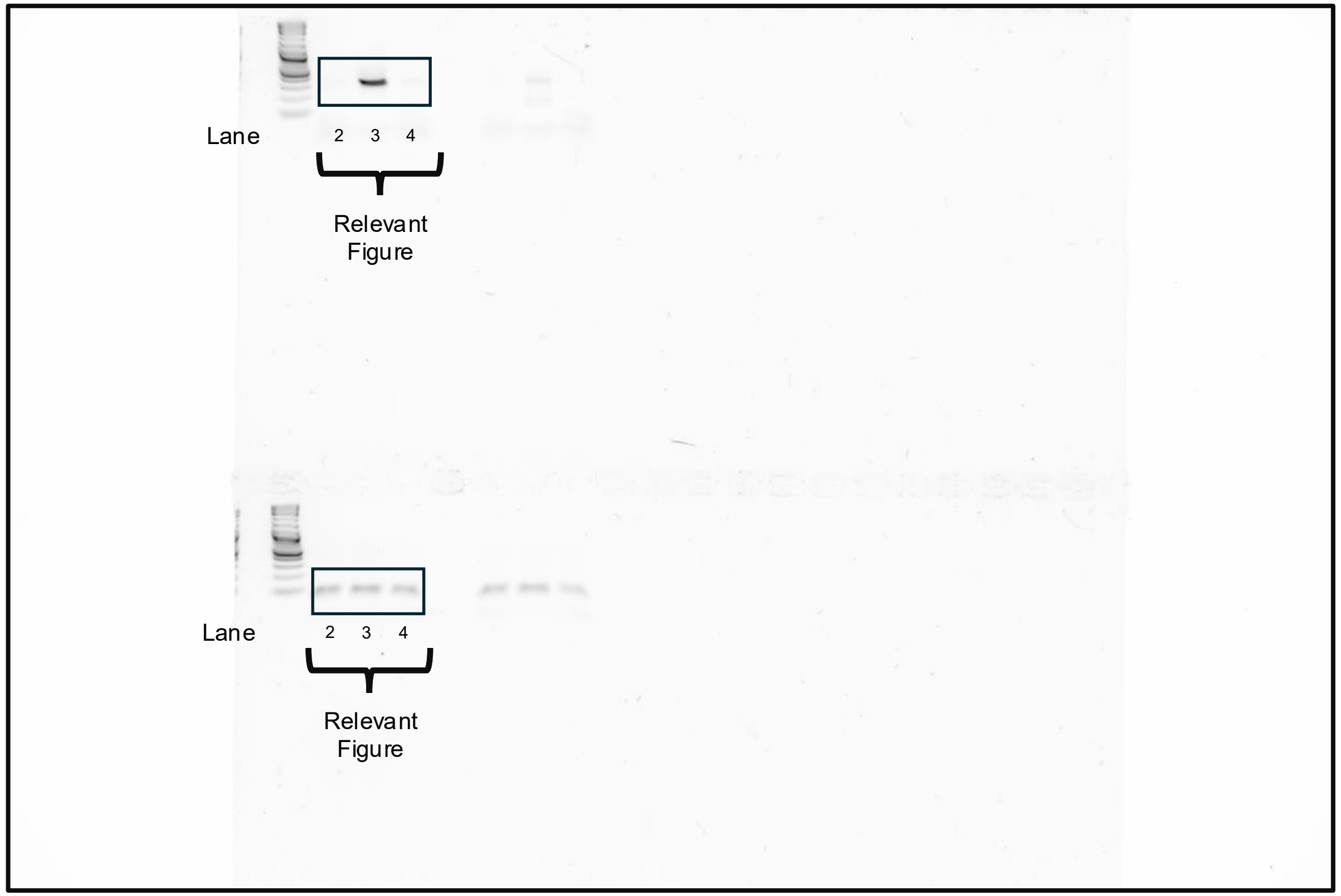

- Full Unedited Agarose Gel for Supplemental Figure 2B
  - Embedded black boxes depict portions of image used in the figure

- Amplified PCR products: IKKβ Transgene (Top Lanes), *Actb* (β-Actin, Bottom Lanes)
  - Lanes 2-4: FACS-Sorted IKKβ^{Tbx4} Mouse Lung Parenchymal cells
    - Lane 2: Epithelial cells
    - Lane 3: Fibroblast cells
    - Lane 4: Endothelial cells
